# Standardised packaging, minimum excise tax, and RYO focussed tax rise implications for UK tobacco pricing

**DOI:** 10.1371/journal.pone.0228069

**Published:** 2020-02-13

**Authors:** Rosemary Hiscock, Nicole H. Augustin, J. Robert Branston, Anna B. Gilmore

**Affiliations:** 1 Tobacco Control Research Group, Department for Health, University of Bath, Bath, England, United Kingdom; 2 Department of Mathematical Sciences, University of Bath, Bath, England, United Kingdom; 3 School of Management, University of Bath, Bath, England, United Kingdom; University of Miami, UNITED STATES

## Abstract

**Background:**

Standardised packaging for factory made (FM) and roll your own (RYO) tobacco was fully implemented in the UK in May 2017. Around the same time, several changes to the tax system were applied (a Minimum Excise Tax (MET) for FM products and tax increases weighted towards RYO products). The tobacco industry claims that standardised packaging will lower prices (a disincentive for quitting) by commoditising the product, yet had itself taken advantage of the previous tax regime to achieve large profits from premium brands while also keeping some products’ prices relatively low. Here we evaluate the impact of standardised packaging, the MET and the RYO focussed tax changes on price and industry profitability.

**Methods and findings:**

Nielsen electronic point of sale (EPOS) data (May 2015 to April 2018) were used to calculate real (inflation adjusted) monthly price per stick overall, by cigarette type (FM and RYO) and by seven market segments. Trend estimation, using additive mixed models, assessed weighted average price (weighted by volume of sales) and tobacco industry net revenue changes. The beginning and end of the data series were compared in terms of: (a) average monthly price growth, (b) average monthly net revenue growth, and (c) undershifting and overshifting patterns after tax changes. FM and RYO real prices changed little over the 3-year period—overall prices rose by about 1p per stick. There was no evidence of commoditisation with prices of all FM segments (but not RYO) rising faster after the implementation of standardised packaging than immediately beforehand. The prices of the cheapest FM brands rose with the implementation of the MET. RYO price increases did not close the gap to FM pricing levels despite RYO focussed tax increases. Tax changes following the implementation of standardised packaging and the MET were more widely and quickly passed on to smokers in the form of higher prices than the tax change pre-implementation. The main limitations are first that because we do not know the exact mechanism by which Nielsen scales up sample data to provide UK estimates, we could only use data for a set three year period during which the same adjustments are made. Second, the tax and standardised packaging events were sometimes too close in time to separate their consequences statistically. Third, tobacco prices may also be affected by external factors such as changes in smokers’ disposable income or availability of electronic nicotine delivery systems.

**Conclusions:**

There was no long-term lowering of tobacco prices after the implementation of standardised packaging as predicted by the industry. The introduction of the MET was successful in increasing the price of the cheapest FM cigarettes and narrowing the price gap between FM brands. The RYO tax increases were, however, insufficient to narrow the price gap between RYO and FM. Overall, undershifting became less extensive indicating that tobacco industry manipulation of the tax system which had previously kept cheap products available had declined. This suggests that standardised packaging and a MET will likely contribute to further declines in UK tobacco use.

## Introduction

Tackling tobacco smoking prevalence through implementing both well-established regulations and new controls, is required to improve human health. Tobacco tax increases are the most effective tobacco control method in terms of reducing tobacco smoking prevalence [1–9], consumption [10, 11], initiation [12, 13] and inequalities in smoking rates [14–17], and have the advantage of raising government revenue [18]. Standardised packaging, a new tobacco control policy, has recently begun to gain traction around the world [19]. This, involves the removal of branding, and mandates the regularisation of other pack features such as colour, font, health warnings, internal packaging, stick design, and sometimes even pack size [19].

The tobacco industry has fought the implementation of standardised packaging in every jurisdiction [20–24]. One of its main arguments in opposing the policy is that it would result in the commoditisation of tobacco and hence reduced prices [25, 26]. As high tobacco prices are key to reducing use, such arguments may deter governments from implementing standardised packaging legislation and it is therefore important to understand their veracity.

The tobacco industry argument that standardised packaging, by commoditising tobacco products, will lead to a fall in tobacco prices [25] might be seen as part of the tobacco industry’s claim that every tobacco control policy will lead to dystopian outcomes [27]. The industry is in fact unlikely to want to cut prices as this would reduce its profits. As the product is highly addictive [28] and price inelastic [29, 30] the need to cut prices is also likely to be limited. On the other hand, the uniform drab pack colour mandated by standardised packaging legislation [31] could reduce the incentive for smokers to pay extra for premium brands and thus average tobacco prices might decline and/or the price range might narrow (commoditisation).

The UK was the second country to implement standardised tobacco packaging following Australia’s lead in 2012 [31]. The UK legislation, in concert with the packaging restrictions stipulated in the EU Tobacco Products Directive [32], came partially into force on 20^th^ May 2016 (when all *newly* manufactured or imported FM and RYO products had to be sold in standardised packaging), with full implementation (when all packs had to be standardised) on 20^th^ May 2017. The appearance of standardised packs was therefore gradual and most tobacco products remained in branded packaging for most of this year-long implementation period [33].

These years (2016 and 2017) also saw a number of changes to tobacco taxation (Box 1 and Table 1) which will have also impacted tobacco prices. Most notable were the introduction of a minimum excise tax (MET) on FM cigarettes in May 2017 and two increases in specific taxes on RYO products in addition to the usual tax escalator (that applied to both FM and RYO taxes). The decision to implement a MET [34], confirmed at the March 2017 budget [35], was driven by evidence of a growing price gap between cheap and expensive FM cigarettes [36, 37]. This was caused by the tobacco industry’s pricing strategy—undershifting taxes (absorbing the tax increase) on its cheapest products while overshifting taxes (increasing its prices over and above the tax increase) on more expensive products in order to maximise profits [36]. The predominant pattern of overshifting has accounted for the tobacco industry’s ability to continue increasing profits despite declining sales [38]. The industry’s fear that this may become difficult with no pack branding would seem to underpin its opposition to standardised packaging.

**Box 1. Tobacco taxes in the UK**

Tobacco is taxed in the UK as follows (see also Table 1): specific (ad quantum) taxes (a tax on quantity) are applied as a fixed amount of tax payable on each unit sold and therefore narrow the percentage price difference between cheap and expensive products. Ad valorem taxes (a tax on value) are paid as a percentage of the pack price and thus raising ad valorem tax rates tends to widen price differences between cheap and expensive products. In the UK both types of taxes are applied to factory made cigarettes (FM), while only specific taxes are applied to roll your own (RYO) tobacco. Both products have an additional ad valorem Value Added Tax (VAT) which is applied to most goods and services in the UK [39].

The ad valorem tobacco tax on FM remained unchanged during the period that standardised packaging was introduced, as did VAT. However, on two occasions around the time of implementation of standardised packaging, specific RYO tax rises were larger, in percentage terms, than those on FM products [40, 41] (Table 1). This aimed to narrow the price gap between FM and RYO tobacco. Previously the price difference between these products had led to growing numbers of smokers downtrading to RYO and a growth in RYO sales [37, 42].

A further change was the implementation, for the first time in the UK, of a minimum excise tax (MET) on FM cigarettes in May 2017, the point of full implementation of standardised packaging [43] (Table 1). The MET requires that if a product’s tobacco tax liabilities do not ordinarily meet a specified floor level then an alternative approach to calculating tobacco duty is utilised such that this minimum tax level is met.

**Table 1:** Tobacco product (FM and RYO) taxation May 2015 to April 2018.

	Budget stipulated tax changes[35, 40, 41, 44–51]			Tax rates[52–54]				Inflation [55]^b^
	All tobacco	FM only	RYO only	VAT^a^ % pack price	FM		RYO	12 month % change
					Ad valorem % pack price	Specific £ per 1000 sticks	Specific £ per kg	
Baseline rates (May 2015)				20.0	16.5	189.49	185.74	0.3
Budget enactment								
16 March 2016	2% above RPI		+3% (total 5%) above RPI	20.0	16.5	196.42	198.10	0.8
8^th^ March 2017	2% above RPI			20.0	16.5	207.99	209.77	2.3
20^th^ May 2017		MET £268.63 per 1,000 cigarettes^c^						2.7
22^nd^ November 2017	2% above RPI	MET £280.15 per 1,000 cigarettes^c^	+1% (total 3%) above RPI	20.0	16.5	217.23	221.18	2.8

^a^VAT (value added tax—a sales tax)

^b^The CPIH (Consumer Price Index including owner occupiers housing costs) was the UK government lead inflation index during this period [56].

^c^MET: MINIMUM EXCISE TAX—From 20th May 2017 the specific rate of duty on cigarettes is an amount equal to the higher of the following alternatives. Either A. £207.99 per 1,000 cigarettes plus 16.5% of retail price, or B. £268.63 per 1,000 cigarettes. From 22nd November 2017 the specific rate of duty on cigarettes is an amount equal to the higher of the following alternatives: Either A. £217.23 per 1,000 cigarettes plus 16.5% of retail price, or B. £280.15 per 1,000 cigarettes [54].

Previous analyses of the impact of standardised packaging on tobacco price are limited and have mixed findings. In Australia, prices continued to rise after standardised packaging was introduced in 2012 in all market segments [57]. However, there were a number of differences between the UK and Australian legislation and the sales data available to Australian researchers was limited [19]. In the UK, the current evidence suggests that prices rose during the year long implementation period (May 2015 to May 2016) and then fell afterwards [58, 59]. Breton et al [59] analysed UK Nielsen data between March 2013 and June 2017, only a month after full implementation. Critchlow et al’s analysis of UK convenience store sales data [58] had a longer follow up period (until October 2017) but did not include the post full implementation tax change (November 2017) and analysed prices of only 20 individual products. Their analysis was descriptive only and thus unable to generate confidence intervals or make inferences about price changes. A more recent paper analysing data to May 2018 suggests that prices may have eventually risen [60]. These contradictions may have arisen due to the failure to take into account non-linear trends. Further studies have been called for [60] to determine whether price rises have been market wide and whether overshifting taxes on more expensive brands continued. All papers failed to account for or examine the complexity of tobacco industry pricing [37] and were focussed on standardised packaging rather than the accompanying introduction of the MET.

To overcome these limitations, our analysis covers a long follow up period, all widely sold products, examines impacts on both price and industry revenue, and benefits from more sophisticated analysis including weighting prices for volumes sold and the use of additive mixed models, a special case of GAMMs [61]. Such models enable us to take account of tobacco industry market segments and geography in order to take into consideration different areas’ price ranges and time trends. In addition, we could model non-linear relationships between price and independent variables, and take into account temporal correlations in monthly prices. In particular, by including market segment specific non-linear effects of time we can model the tobacco industry reaction (immediate and latent) to the different tax events, the introduction of standardised packaging, and other unexplained phenomena that could be influencing price. This is preferable to a breakpoint approach, since there are many potential breakpoints in the data and the tobacco industry’s reaction to legislation (tax, minimum packsize, plain packaging) is not necessarily immediate. Therefore, the optimal placing of breakpoints is unclear. Thus, our modelling choices allowed us to fit smooth trend lines that allow the trends to vary by month. Furthermore, we were able to estimate confidence intervals in order to understand whether patterns in the data are noise or evidence of a significant change in retail prices. We also took advantage of detailed knowledge of tobacco industry pricing strategies [9, 36–38, 62–66] to aid analysis, design, and interpretation of model results.

Our paper also provides the first analysis of the impact of the MET in the UK. Although a number of countries have implemented tax or price floors there is currently little evidence on whether a MET is effective in raising the minimum price of tobacco [18, 67, 68]. A recent review of the effectiveness of minimum floor prices identified just four studies [68], three of which were hypothetical modelling studies and the fourth an observational study which suggested that sales of legal cigarettes below the minimum price declined after implementation in Malaysia. A separate evaluation did consider the implementation of an MET on FM cigarettes in Spain, [69, 70] suggesting that the MET did increase the minimum price and compressed the FM price distribution but left the ability to switch to RYO products available and consequently had no impact on smoking prevalence. However, Spain has not implemented standardised packaging to date and has lower tobacco tax levels than the UK [71]. A study of the Ukraine suggested that sales declined and tax receipts rose after the implementation of a minimum total excise tax in 2008, but several other tax changes were also implemented simultaneously so the impact of the MET remains unclear [72].

Our previous analysis of a 2012 tax change intended to reduce the price gap between FM and RYO products showed no evidence of long-term effectiveness. The tobacco industry increased the prices of premium FM products markedly (increasing the average price per stick) whilst increasing the numbers of the cheapest RYO products on the market, whose prices remained flat in real terms [37]. Here we have two further RYO focussed tax changes to analyse which occurred before and after major changes to pricing and branding via the implementation of standardised packaging and the MET.

In light of the complex changes to UK tobacco control policy detailed above, this paper addresses the following questions:

1. Is there is any evidence that commoditisation happened after the introduction of standardised packaging as suggested by the tobacco industry:
  - Did overall tobacco prices fall following implementation of standardised packaging?
  - Was there a decline in FM prices leading to a decline in the price gap between FM and RYO tobacco types?
  - Was there a decline in FM premium prices leading to a decline in the price gap between the most expensive and cheapest FM cigarettes?
2. Was the MET associated with a rise in the price of the cheapest FM cigarettes and a narrowing of the price gap between these and other FM cigarettes?
3. Were the RYO focussed tax rises associated with a rise in price of RYO tobacco and a narrowing of the price gap between RYO and FM?
4. Did the tobacco industry pricing strategy and the extent of over- and under-shifting of taxes change with introduction of standardised packaging and linked tax changes?

## Methodology

### Data

Nielsen, a global information company, collates electronic point of sales (EPOS) data on tobacco sales from nearly 90% of UK supermarkets (including a census of sales from stores owned by the largest four UK supermarket chains) and a stratified sample of 15% of convenience stores [73]. Nielsen then scale up the sales and pricing data to population level to develop estimates for all UK shops selling tobacco products. As the scaling up is undertaken for 3-year periods we took a single 3 year dataset in order to avoid Nielsen’s possible changes in scaling procedures mid-way through our period of analysis. Strata for the scaling up are based on geography, shop/group type, and fascia (owner or chain) [74, 75].

The study was given ethical approval by the Head of Department of the University of Bath Department for Health, consistent with University of Bath guidelines for studies without human participants. The data comprised of product sales rather than human participants, thus participant consent was not needed. Product names and geographies were provided but Nielsen does not provide the names of stores/fascia selling the products to protect market sensitivities.

Monthly data were available on volume of sales, sales prices, and distribution of sales for each product (or stock keeping unit (SKU)) for sales within 11 geographical areas. There were 883 836 monthly observations. For sample design reasons, Nielsen recommend only analysing observations of widely distributed SKU (sold via 10% or more retailers). We therefore excluded monthly observations where distribution information was missing (164 monthly observations) and monthly observations that did not reach this threshold either in the UK overall, or within a given geographical area if sales did not reach the threshold in that area. There were 107 571 monthly observations in the final models (91% of the original volume of sticks).

### Variables

#### Price (dependent variable)

To allow for the variation in pack size over time (7) we used price per stick (FM) or stick equivalents (RYO). For the latter we used a weight of 0.5g per stick based on the latest evidence on RYO cigarette size in the UK [66]. Real prices were calculated via adjusting nominal prices for inflation to May 2015 prices using the official UK measure of inflation (see Table 1).

#### Net revenue (dependent variable)

We calculated ‘net revenue’ per stick as the price per stick minus the taxes due on each stick [37, 76]. Thus net revenue is tax exclusive prices. The taxes were the tobacco taxes (specific, ad valorem, and MET) and VAT. This represents the amount of money available to the tobacco industry after all taxes have been paid. This figure will cover manufacturing, packaging, retailing, and distribution costs with the rest accruing as industry profit. Net revenue therefore gives an estimate of tobacco industry profitability given that most of net revenue is tobacco industry profit as manufacturing costs are low and little profit goes to retailers [77, 78].

#### Time

To understand time trends in pricing, time (in months) was included in the model. There were 36 months in the analysis from May 2015 to April 2018.

#### Market segment

Market segment was included as an independent variable because the tobacco industry groups products into segments with different pricing strategies for each [36, 37]. Market segment names (FM premium, midprice, value, and subvalue, and RYO premium, midprice, and value) and brand variant allocation to those segments were based on our previous comprehensive review of the commercial literature and linked analysis of Neilsen data [37] (see Table A in S1 Appendix for derivation of brand variant). We used both graphical representation of pre-legislation pricing (May 2015 –April 2016) and an updated review of the commercial literature to check this allocation. Three of 348 brand variants were moved into different market segments based on the majority time spent in each market segment identified via the graphs.

A few SKUs (5% of RYO and 0.1% of FM by volume) could not be classified by market segment so were grouped into a “no segment” category. The majority of these were RYO ‘combi packs’ that included rolling papers and filters within the pack.

#### Geography

Nielsen sales data were historically designed to aid commercial decisions on purchasing television advertising time. Thus UK sales data are provided for 11 geographical areas based on commercial television (ITV) transmitters (areas covered by more than one station are allocated to the station most used by their population) (See Fig A in S1 Appendix). Geography was included as an independent variable in our model because different areas have different traditions of tobacco use, both in prevalence of use and tobacco type [9, 79]. In addition, the devolved nations of the UK have some control over tobacco policy and English regions, which approximately map onto transmission areas, have a history of local tobacco control bodies which vary in their approaches, funding, and impact. The inception of smoke free prisons and the bans of smoking with children in cars, proxy purchasing for children, and selling e-cigarettes to children varied between England and the devolved nations during this period [80, 81].

### Statistical analysis

#### Multivariable models

Exploratory analysis showed a strong temporal correlation of monthly price observations within SKU—that is the price in one month was very similar to the previous month’s price, and also a non-linear relationship between price and time (Fig B in S1 Appendix). Thus a linear model with ordinary least squares (OLS) estimation was inappropriate here. Model requirements were met via an additive mixed model, which is a special case of a generalized additive mixed model (GAMM) [82, 83]. The model was mixed because an autoregressive process for residuals [84] was assumed. Diagnostic residual plots [83] illustrate that our final model was a good fit (see Fig D in S1 Appendix). Models were estimated using R version 3.5.0 and the mgcv package [85]. Parameter estimation used penalised (iteratively reweighted) least squares.

We first modelled monthly price per stick (*pps*) of stock keeping unit (SKU) (at a particular month and geographical area) *i* (thus each case in the analysis was a SKU at a particular month and geographical area) as:

$$\begin{array}{c} pps_{i}=\alpha +f_{k}(t_{i})+\beta_{j}+\gamma_{k}+\epsilon_{i} \end{array}$$

where *pps*_*i*_ with i = 1, …,107 571 is observed at *time t*_*i*_, *geography j*, and *market segment k*. *t*_*i*_, represents the continuous variable time and *f*_*k*_(*t*_*i*_) are non-linear functions of time for each of the market segments k. *β*_*j*_ and *γ*_*k*_ represent the fixed effect parameters for the categorical variables geography and market segment respectively. The subscript *j*, indicates that the equation applies to cases located at any of the 11 geographical areas (Central England, East of England etc.) thus j = 1, …,11 and the subscript *k* indicates that the equation applies to cases in any of the eight market segments (FM premium, FM midprice etc.) thus k = 1,…,8. Both of these fixed effects are coded as dummy variables, with the first category being the reference category (see Table B in S1 Appendix). The error *ϵ*_*i*_ is assumed to have a normal distribution with mean zero and a covariance matrix reflecting the assumed auto-regressive correlation structure in time within SKU by Geography (see below).

Our second model was identical to the first model but substituted real net revenue (see below) for real price per stick:

$$\begin{array}{c} {net\;revenue}_{i}=\alpha +f_{k}(t_{i})+\beta_{j}+\gamma_{k}+\epsilon_{i} \end{array}$$

*Addressing the lack of independence of monthly observations*. There was a very strong positive correlation between a SKU’s price per stick and the previous month’s price per stick (an estimated positive temporal correlation of errors *ρ* = 0.98). Ignoring this correlation would have led to a negative bias in the variance estimates and hence too narrow confidence intervals. We addressed this by assuming an autoregressive process of order 1 (AR1), that is the current error is correlated with the previous month’s error with correlation *ρ* within a SKU. Assuming an AR1 produced standardized residuals which were not correlated in time (as they would otherwise have been—see Fig C in S1 Appendix).

We could have accounted for correlated temporal errors within a SKU by geography with a random effect for SKU (or SKU in a particular geography) but instead we used a first order autoregressive process (AR1) as the obvious model choice for time series data because it takes into account the temporal order of residuals.

There was a similar strong positive correlation between a SKU’s net revenue per stick and the previous month’s net revenue per stick. Thus an AR1 was assumed for both models.

*Addressing the non-linearity of time trends*. Exploratory analysis showed that the time trends of average weighted price and net revenue in time were non-linear (see Fig B in S1 Appendix). For modelling these non-linear time trends a linear model could be used if breakpoints in the regression lines were included for the different tax events [86]. However, since there were many potential breakpoints in the data, we used the more flexible GAMM model, thereby allowing the data to estimate the form of the time trend.

The terms *f*_*k*_(*t*_*i*_) were non-linear functions of time for each of the market segments k represented using penalized regression splines; in particular we used a cubic regression spline basis. The terms represent any effect of time on price per stick (pps) or net revenue as a result of tobacco industry reacting to the different tax events, the introduction of plain packaging, minimum pack size, and other pricing strategies.

Model selection (using Akaike information criterion (AIC) [87]) showed that including an interaction between time and market segment via smooth functions of time varying by market segment *f*_*k*_(*t*_*i*_) was preferable to an equivalent linear model. The AIC statistics for both *pps* (linear model = -861505 and smooth model = -876178) and *net revenue* (linear model = -891576 and smooth model = -915533) were lower (more negative) for the smooth models. This means that the smooth models have a better goodness of fit compared to the linear model and are hence the preferred choice here.

There were significant differences in *pps* and *net revenue* intercepts by geography but no evidence that the shape of time trends differed by geography (see Fig B in S1 Appendix). Thus main effects of geography were modelled but not interactions.

#### Trend estimation using model results

To examine prices over time, we present temporal trends graphically as models including smooths do not have a linear relationship between the dependent and independent variables so we cannot rely on model coefficients to understand the nature of the association. We used modelled price to calculate weighted average (mean) prices (WAP -weighted by sales volume) per stick overall, by tobacco type (FM and RYO), and for each price-segment. To calculate the WAP, the number of sticks sold for each SKU was used to create the market share, and the applicable market shares were then multiplied by the SKU’s price and summed. The methodology for estimating confidence intervals is based on Bayesian simulation of the model coefficients [85 (section 7.2)]. We show the estimated overall average (mean) price with Bayesian confidence intervals. We plot confidence intervals so that we know whether changes are likely to be meaningful or just the result of natural variation (i.e. noise). We used a similar process to calculate average net revenue. More details are given in S1 Appendix.

**Fig 1:**
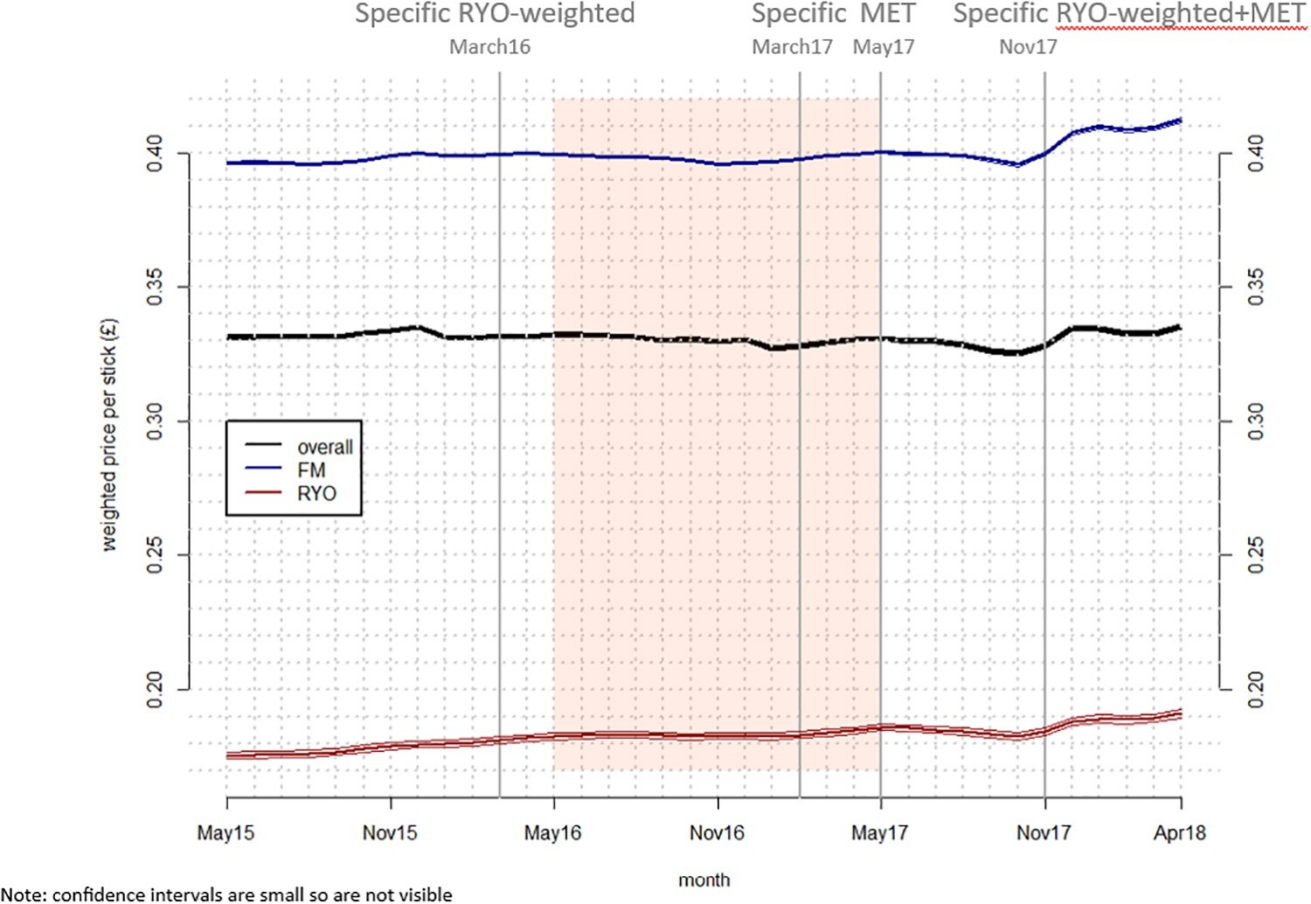
Mean and 95% confidence intervals (CI) of weighted real price per stick overall and by product type (vertical lines denote tax changes and the shaded area is the implementation period).

**Fig 2:**
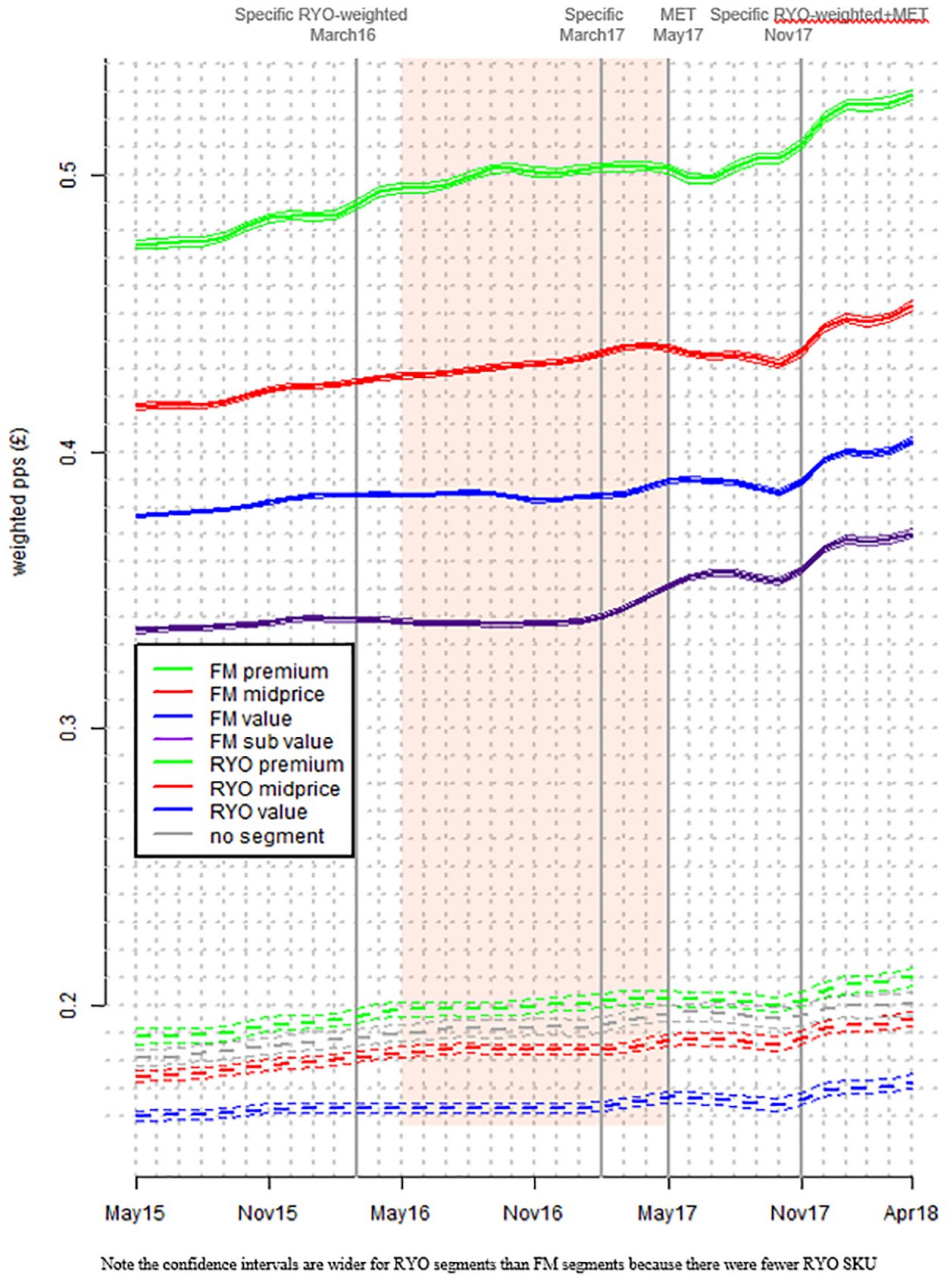
Mean and 95% confidence intervals (CI) of weighted real price per stick by (a) FM market segment and (b) RYO market segment and no segment (vertical lines denote tax changes and the shaded area is the standardised packaging implementation period).

**Fig 3:**
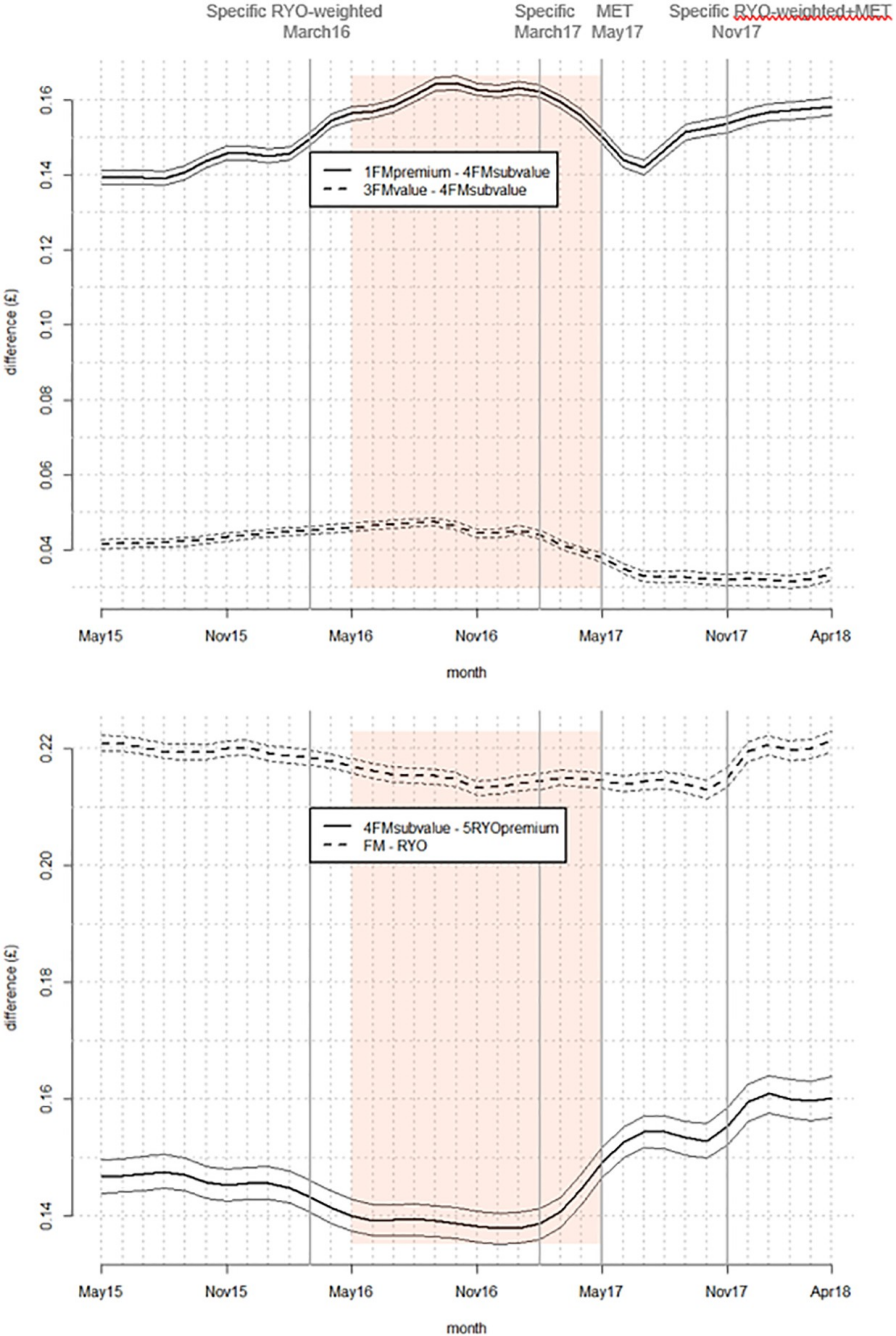
Gaps in weighted average real price per stick between (a) selected FM market segments and (b) FM subvalue and RYO premium and FM and RYO overall (vertical lines denote tax changes and the shaded area is the standardised packaging implementation period).

First we examined WAP over time overall, and then by tobacco product type, and by market segment (Figs 1 and 2). Then we examined the difference in weighted average price between product types and segments (the ‘price gap’–Fig 3) by subtracting the price of the cheapest product from the more expensive. Comparisons were made between: FM premium and FM subvalue; FM value and FM subvalue; FM and RYO; and FM subvalue and RYO premium. In these sets of figures, vertical lines denote tax changes and the shaded area the implementation period. Where 95% confidence intervals do not overlap, differences are significant. Note that in Figs 1 and 2a the 95% confidence intervals are hard to see in some cases because the intervals are very narrow.

**Fig 4:**
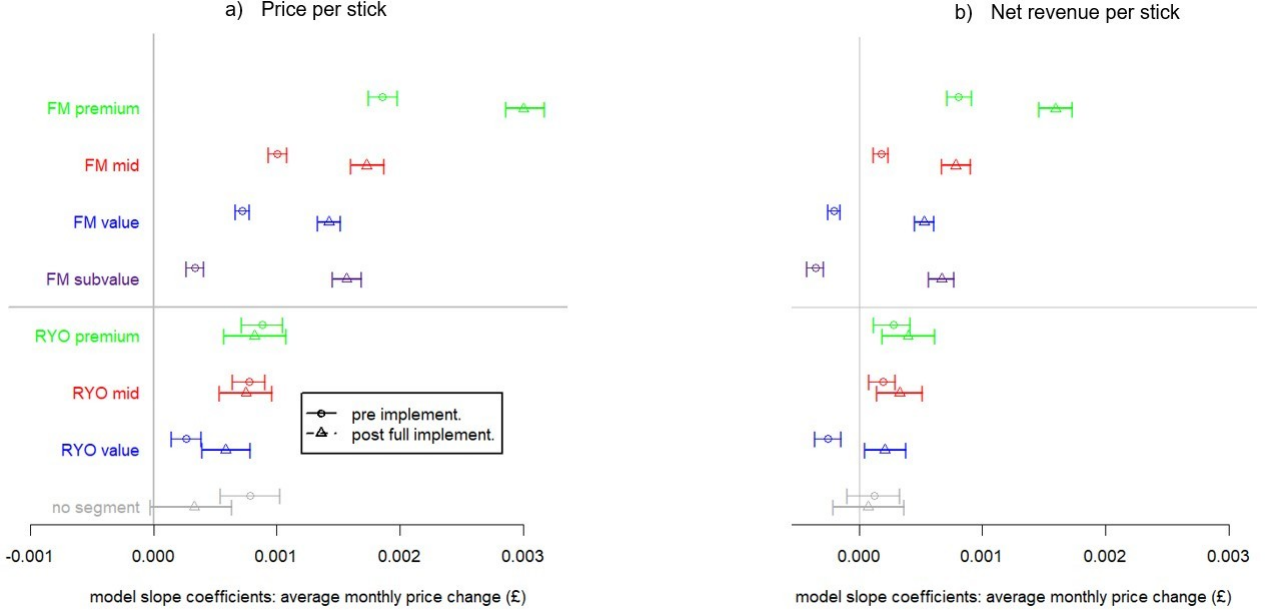
Forest plots of the pre- and post- implementation periods (June to March 2015/16 and 2017/18 respectively) showing mean monthly change in (a) weighted average price per stick and (b) weighted average net revenue per stick.

To provide further clarity we explored whether patterns of pricing and net revenue changed pre- and post-legislation via forest plots comparing average monthly change (in £) of (a) price, and (b) net revenue over ten months before and the same ten calendar months after the introduction of standardised packaging and MET (Fig 4). The same months (June to March) were used to reduce the impact of seasonal variations.

**Fig 5:**
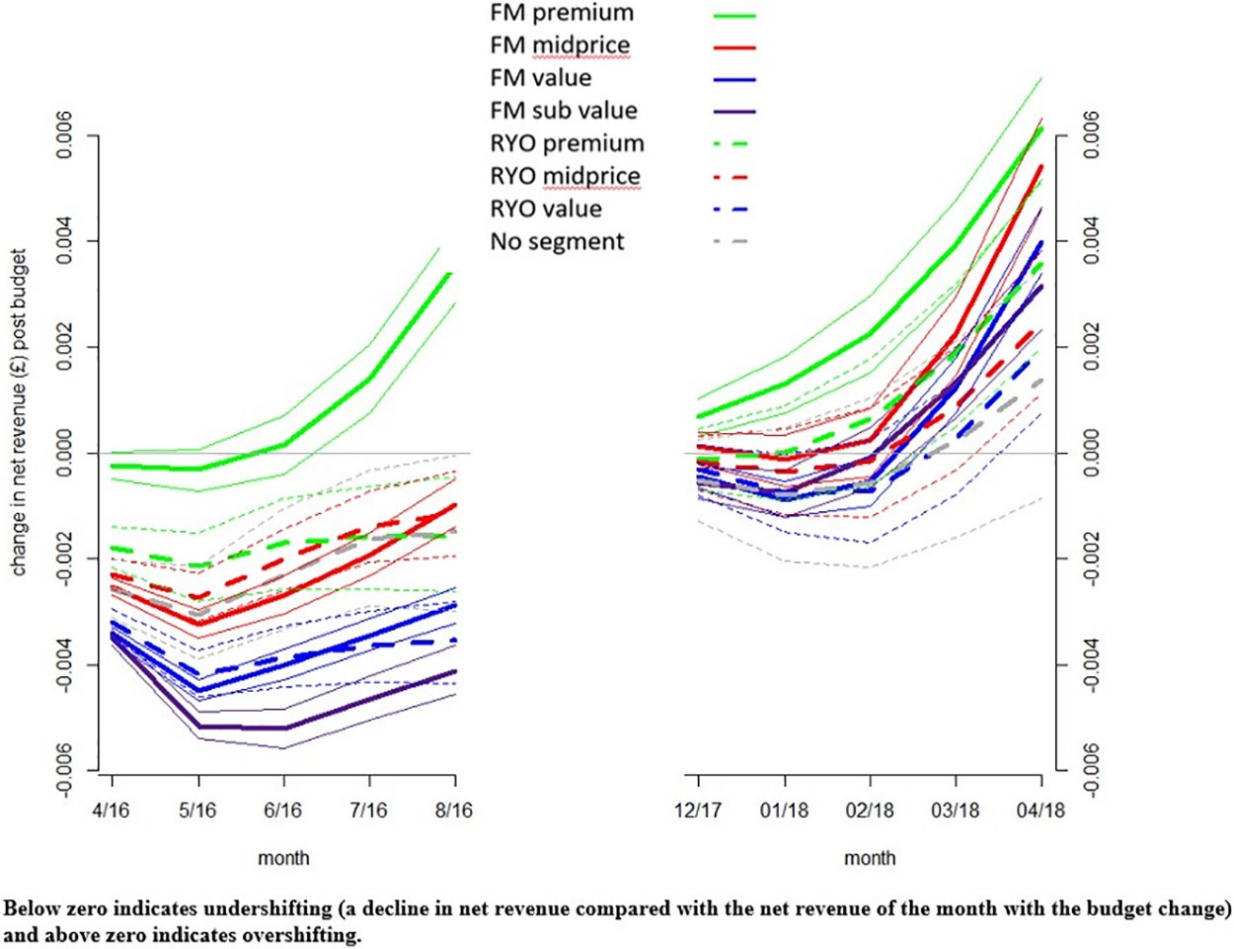
Mean change in weighted average net revenue (& 95% CI) of market segments during five months after (a) the pre implementation tax change (March 2016) and (b) the post full implementation tax change (November 2017).

We then examined changes in net revenue over a five month period, after both the first and final tax changes in the data series, to examine whether patterns of undershifting and overshifting of taxes changed (Fig 5). Follow-up was undertaken for five months because this was the length of the data series after the final tax change. To estimate the change in mean net revenue post tax change, the net revenue per stick (in pence) for the month with the tax increase was subtracted from the net revenue for each of the following five months. Below zero indicates undershifting (a decline in net revenue compared with the net revenue of the month with the budget change) and above zero indicates overshifting.

## Results

### A) Standardised packaging/commoditisation

The overall average (mean) weighted real price per stick increased significantly between the first and final months in the data series (May 2015 and April 2018) but by only 0.4 pence (Fig 1 and Table E in S1 Appendix). Across the same period both FM and RYO prices rose by 1.6 pence. Analysis by market segment shows that FM premium price growth between the beginning and the end of the data series was higher (five pence) than the other market segments (between four and one pence) (Fig 2 and Tables F and G in S1 Appendix). The price gap graph (Fig 3) shows that the gap between FM premium and FM subvalue varied over time but was again growing by the end of the data series. The gap between FM and RYO prices (Fig 3) was similar at the beginning and end of the dataset—having declined slightly and then returned to previous levels within the data period.

The forest plot (Fig 4a) shows that post full implementation of standardised packaging, FM premium product prices were growing significantly faster than all other market segments and furthermore were growing significantly faster than pre-implementation (see also Table H in S1 Appendix).

In summary, there was no evidence of any long term narrowing in price gaps as a consequence of falls in prices. Indeed, rather than showing price declines, premium products continued, overall, to have marked price rises compared with other price segments. Thus there was no evidence of commoditisation.

### B) MET

There was a marked increase in the cheapest FM market segment (subvalue) between March 2017 when the MET was announced and July 2017, two months after full implementation of standardised packaging. During this period FM subvalue prices increased significantly by 1.3 pence. The only other market segment to increase significantly during this period was FM value which only increased by 0.5 pence (Fig 2 and Tables F and G in S1 Appendix). In fact, the price gap graph (Fig 3) shows that during this period the gap between FM value and FM subvalue decreased significantly and this reduced gap was maintained for the remainder of the data series.

The forest plot (Fig 4a) shows that FM subvalue price growth pre and post MET implementation was significantly lower than in other FM market segments but post-implementation price growth had increased and was not significantly different from the FM value and midprice segments. In line with this, the difference in price growth for the FM subvalue segment across these two periods was larger than that of the FM value and FM midprice segments.

In summary, there was marked growth in the price of the FM subvalue segment that occurred around the time of the implementation of the MET and that outstripped growth in other segments. Pricing and price growth of the FM subvalue products subsequently became more similar to FM value products.

### C) RYO tax rises

RYO and FM pricing patterns were similar after the RYO focussed tax rises: there was little change after the March 2016 tax change but a significant change after the November 2017 change. Moreover, the size of the increase after the November 2017 tax change was larger for FM than RYO: 0.8 pence for FM and 0.4 pence for RYO (Figs 1 and 2 and Table E in S1 Appendix). At these times there was no narrowing in the price gap between FM and RYO overall or between the cheapest FM segment (FM subvalue) and premium RYO (Fig 3).

There was no significant increase in price growth for RYO premium and midprice brands at the beginning and end of the period studied (June 2015 to March 2016 versus June 2016 to March 2017 (Fig 4a)). Although there was a significant but small increase in the price growth of RYO value brands (.03 pence), this growth was smaller than for all FM segments (see Table H in S1 Appendix).

In summary, increases in RYO taxation have not been associated with marked rises in RYO price per stick that were not observed for FM. There was no preserved narrowing in the price gap between tobacco types.

### D) Changes in patterns of overshifting and undershifting

Specific tax increases in March 2016 and March 2017 were associated with little price change but prices increased markedly after the November 2017 tax rise (Figs 1 and 2). Such price impacts cannot be explained by differences in the nature of those tax increases implying that the differences might be explained by industry pricing and changing patterns of under- and over-shifting.

This contention is supported by data on net revenue. The net revenue forest plot (Fig 4b) shows that pre-implementation the tobacco industry revenues were falling (implying undershifting taxes) in the cheapest FM and RYO market segments (FM value, FM subvalue and RYO value). Post full implementation net revenue was growing in all named market segments so there was no evidence of undershifting. In addition, growth in industry revenue was significantly higher in the post-implementation period in all FM segments and in the value RYO segment. Thus revenue growth tended to be higher after the implementation of standardised packs than before and in no segment did it fall. Notably, net revenue was growing fastest for the FM premium market segment in both periods suggests that the tobacco industry was still able to increase their profits on premium brands.

Tax pass through can be further understood through examining change in net revenue after a tax rise (Fig 5). After the pre-implementation March 2016 tax change, net revenue fell for all segments (the tax changes were undershifted) with the possible exception of FM premium. There were significant differences in undershifting within tobacco type with more undershifting in the FM and RYO cheaper segments. Only FM premium net revenue recovered during the five month follow up period with the tax change being overshifted in the last two months. By contrast in November 2017, following the tax change and the introduction of the MET, the net revenue of FM premium was never undershifted, and the extent of undershifting was far less pronounced—mostly confidence intervals overlapped for all other FM and RYO segments. All market segments recovered within the follow up period (thus there was later overshifting on all products).

In summary, at the beginning of the data series there was evidence of overshifting on the most expensive FM premium products and undershifting on other products—particularly the cheapest FM and RYO market segments. After implementation of standardised packaging, a MET and two RYO focussed tax rises there was little evidence of long term undershifting and overshifting was more extensive. Thus, the tobacco industry manipulation of the tax system which had previously kept cheap products available had declined.

## Discussion

It appears that the tobacco industry paused its usual price raising strategies while standardised packaging was introduced. Only by the end of the follow up period (11 months post full implementation) were prices increasing again and thus more consistent with previously observed trends [37, 42]. The relatively stable price per stick in most market segments for the clear majority of the data series was different from previous years where stick prices were consistently increasing [37] and may explain why the fall in smoking prevalence stalled during this period [88, 89]. Standardised packs were often introduced with similar or lower prices than the variant’s branded SKU before later price rises. The low initial price may have been to attract smokers to buy the new style packs. However, rises in price per stick by the end of the period suggests this has been temporary hiatus in stick price rises and that the new regime of excise tax and MET should ensure future rises.

An idiosyncratic marked rise in price of the cheapest FM cigarettes (in the subvalue segment) occurred around the time that the government announced the introduction of a MET and its implementation. Furthermore, this increase in stick prices was maintained suggesting a long term effect. Although we cannot say that the MET definitely caused the increase, there is certainly a temporal association suggesting it was the likely cause.

Despite RYO focussed tax rises, RYO rises in prices per stick did not close the gap between FM and RYO prices per stick. However, standardised packaging included a RYO minimum pack size of 30g. Prior to implementation smaller pack sizes, such as 12.5g [37], were popular so many RYO smokers were faced with considerable pack price rises during this period. It is therefore possible that economies of scale offered by these larger pack sizes offset the impact of higher RYO taxation. Nevertheless, the growing gap in price per stick between the cheapest FM brands and RYO will need to be addressed in future in order to reduce incentives to downtrade to RYO (instead of quitting).

There were indications that the tobacco industry could not manipulate prices as much as previously. Towards the end of the data series they passed on much of the post full implementation tax rise directly and relatively quickly to smokers (more quickly than in the past). By the final tax rise undershifting on the FM value, FM subvalue, and RYO value brands declined. This implies that by the end of the period studied, the tobacco industry was less likely to keep cheap products available by lowering its net-revenue. It is unclear whether this is the result of policies implemented during this period, or another reason. Nevertheless, if this pattern continues, all FM smokers are likely to be subjected to price rises in the future when taxes increase—not just smokers of relatively more expensive products. FM premium prices continued to grow more rapidly than other products so the gap between premium FM products and cheaper products remains. However, it might not be possible to continue this pattern indefinitely.

Nevertheless, some of the possible price changes with potential public health benefits had not materialised by the end of the data series: indeed, price differentials between premium and cheap products continued to grow. The manifest continued ability of the tobacco industry to increase the price gaps between FM premium and cheap FM, and between cheap FM and RYO products, imply there is potential for further specific tax rises particularly for RYO products and in the MET. A mechanism for a routine increase in MET levels (in place in other EU countries [18]) could be considered, such as linking changes in the MET to previous weighted average prices in order to consistently rachet up prices at the lower end of the market. The availability of alternative, non-combustible, nicotine products assuages concerns about the cost implications on those unable or unwilling to quit the use of nicotine.

### Policy implications

It seems important that standardised packaging is introduced in the context of a regime of tax increases [57, 58] because the tobacco industry in the UK was able to hold down prices post standardised packaging of most products until faced with a tax rise. Nevertheless, the UK experience shows that standardised packaging can be implemented without tobacco prices falling long-term. It should therefore be considered in more countries. The UK experience also suggests that an MET is very plausibly linked to a reduction of availability of cheap tobacco, and hence it should be more widely considered. Finally, the tobacco industry was able to keep low prices per stick for RYO tobacco despite tax rises thus further specific tax rises in the UK are clearly needed.

### Strengths and limitations

Standardised packs were introduced during the implementation period between 20th May 2016 and 20th May 2017. In 2016 and 2017, there were also four tax changes. Given the late appearance of standardised packs in the implementation period and removal of non-compliant SKU (Figure A in S1 Appendix and [33, 59]), it is not possible to distinguish statistically between effects of standardised packaging, minimum pack size, and taxation changes. In addition, FM subvalue prices rose as soon as the MET was announced rather than when it was implemented. We cannot definitively proscribe a cause to this rise as other things were also occurring during this period (a tax change and active transition to standardised packaging). Thus our analysis generally focussed on differences at the beginning and end of the analysis period rather than before and after each change.

Nielsen use electronic point of sale (EPOS) data to provide estimates of the UK tobacco market. The advantage of Nielsen data is that it is based on a census of sales at stores owned by the big four UK supermarkets. Nielsen estimate that 70% to 80% tobacco sales are from convenience stores where a smaller proportion are owned by the big four supermarkets. However, Nielsen estimates of the UK entire grocery market lie within estimates from other sources [90, 91] suggesting they are reasonable. It should however, be noted that our dataset did not cover all tobacco related sales. For example, it did not include sales of: RYO rolling papers and filters (unless sold within tobacco pouches), pipe tobacco; cigars and cigarillos; and sales from specialist tobacconists. Tobacconists revenue is only £454 million per year (under 2% of total FM & RYO sales) and has declined by 9% (2013 to 2018) [92], pipe tobacco is now about 0.6% of total tobacco sales [93], and cigar and cigarillo sales (0.8% of the total [93]) are thought not to be growing in response to standardised packaging [94].

Nielsen have not provided details of how they scale up the sample data to the population level, and hence, for example, whether or not they use modelling. We do not know the exact mechanism by which Nielsen scales up sample data to provide UK estimates, and hence we could only use data for a set three year period during which the same adjustments are made. They do suggest excluding low distributed products for sampling reasons. This has meant our analysis covers only about 90% of the UK cigarette market. Nevertheless we expect to cover the main patterns and we do not believe there is a more comprehensive dataset available.

We have illustrated above that despite Nielsen creating UK wide estimates of the tobacco market, there are various sources of uncertainty, including errors associated with the provided prices. To more fully understand the market we were able to use econometric modelling, with our model explaining 98% of the variance in observed price per stick. Most of the variability in the model is explained by segment and time. Our choice of GAMM modelling is supported by better goodness of fit compared to a linear model. Furthermore our choice is supported by consistencies firstly between price patterns from model output (Fig 1) and the raw data (presented in Figs B1 and B2 in S1 Appendix) and secondly between GAMM models and linear models (Table C in S1 Appendix). For example, all the linear model slopes for market segments are positive, with the steepest slope for premium and the intercepts for region and market segment are also consistent with each other.

Tobacco prices may also have been affected by changes external to the cigarette market in this period. For example, analysis of Euromonitor data suggests that sales of vaping products (e.g e-cigarettes) grew [95]. Beyond the immediate tobacco market, some disadvantaged smokers (analysis suggests more smokers are disadvantaged than the general population [79]) were having incomes squeezed by benefit freezes, benefit payment delays with the introduction of universal credit (a new income benefit), and growth in housing private rental costs [96, 97]. Tobacco industry pricing decisions may have been informed by this squeeze in disposable incomes.

In this paper we have looked at stick level changes. The introductions of the RYO minimum packsize of 30g, when previously the most popular packsize was 12.5g [37], also meant a substantial pack price increase facing many RYO smokers during this period. In addition, we did not look at whether the stable FM subvalue and RYO value pack prices previously reported [37] were able to continue after standardised packaging. However, given the ending of shrinkflation opportunities and price marked packs with standardised packaging legislation, it is unlikely that stable pack prices could have continued.

### Summary

In summary, the implementation of standardised packaging has not led to a long term decline in cigarette prices. By the end of the period there were price rises even in the cheapest FM and RYO products and undershifting on these products was minimised. Thus, it is likely that the combination of standardised packaging, increasing tax liabilities for RYO, and the introduction of the MET had led to pricing changes with potential to reduce smoking rates and thus improve public health.

## Supporting information

S1 Appendix Standardised packaging, minimum excise tax, and RYO focussed tax rise implications for UK tobacco pricing: Supporting information. (DOCX)
