# Supplementary material for: Standardised packaging, minimum excise tax, and RYO focussed tax rise implications for UK tobacco pricing
Source: PLoS One. 2020 Feb 13;15(2):e0228069. doi: 10.1371/journal.pone.0228069 (PMC7017998; doi:10.1371/journal.pone.0228069)
Supplement: S1 Appendix — (DOCX) [file pone.0228069.s001.docx]

Appendix

Standardised packaging, Minimum Excise Tax, and RYO focussed tax rise implications for UK tobacco pricing: Supporting Information

# Contents

Description of cases in the analysis

Description of information provided for each SKU

Description of tax calculations

More detail on how time was modelled in GAMM and time trend estimation

Regression results

Modelled stick prices overall by tobacco type and market segment

R code for gamm fitting and trend estimation

# Description of cases in the analysis

The median number of SKU sold in each geography in the first and last months of data (May 2015 and April 2018) were 1133 (range 947 to 1230) and 388 (range 280 to 558) respectively (see fig S1). There were 9006 regional entries of SKUs widely distributed in at least one month of the 36-month analysis period. On average SKUs were sold widely for 12 months so a third of the period. The number of SKUs declined because standardised packaging necessitated the withdrawal of price marked SKUs and SKUs where products were sold in small packs. The graph shows the main withdrawal period was between April and August 2017.

Figure A Number of SKUs sold over time by geography


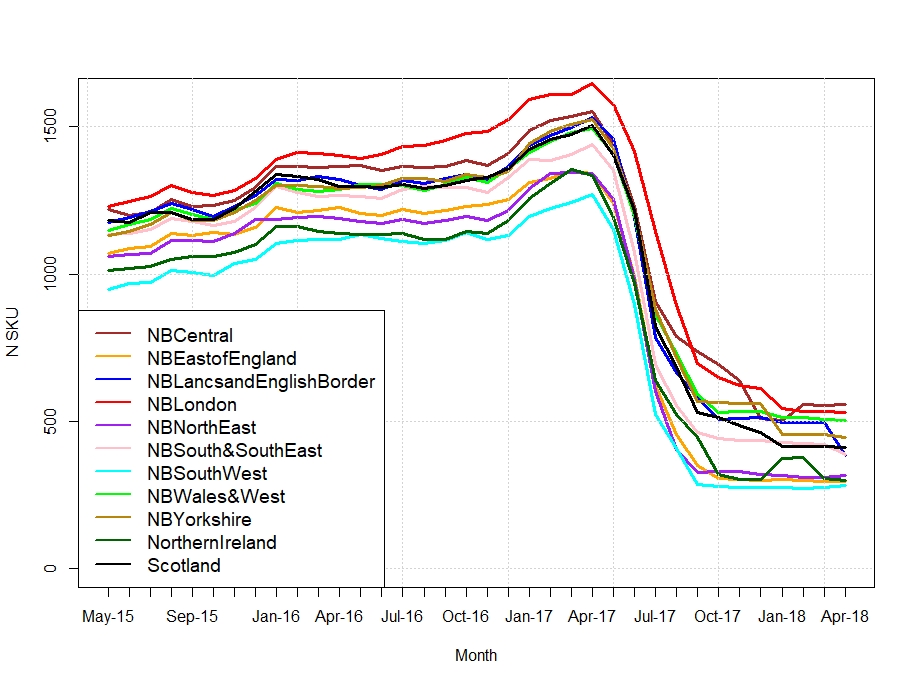

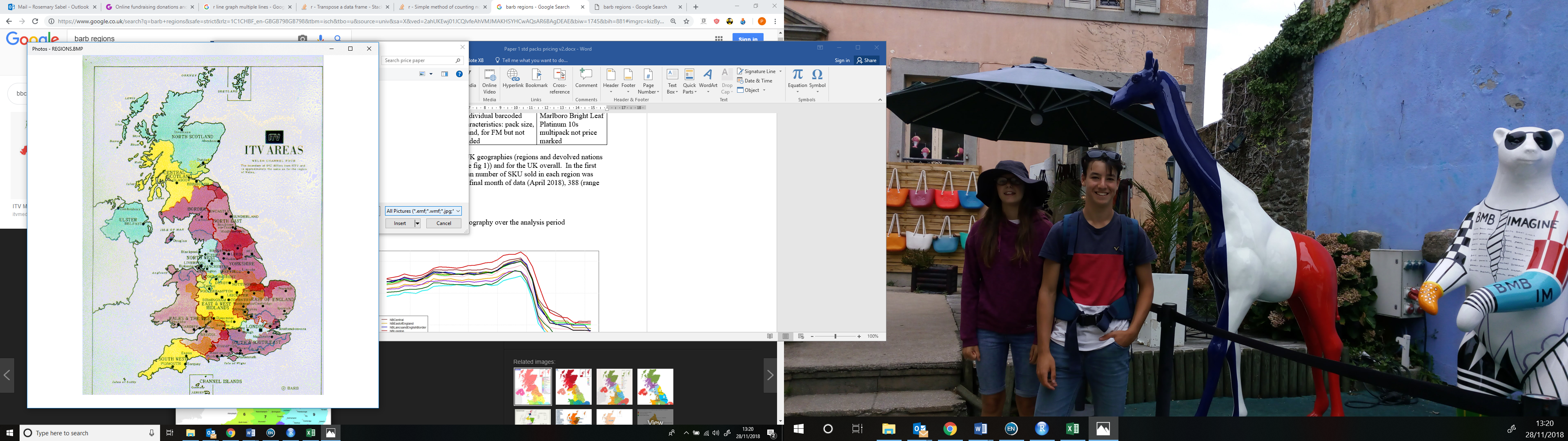


# Description of information provided for each SKU

Using a hierarchical structure, Nielsen records for each SKU - the tobacco brand, brand family, brand variant (table 2), and then specific features of the pack (e.g. size (number of sticks (FM) or weight of contents (RYO)), whether packaging is pricemarked or (for FM only) standardised, whether sold as a single or multi-pack).

Table A Hierarchical brand architecture available in the Nielsen data with example

| BRAND | Marlboro |
| --- | --- |
| BRAND FAMILY Products with the same brand name but sold at different price points | Marlboro Bright Leaf |
| BRAND FAMILY VARIANT Product at same price point but with different length (e.g. superkings, kingsize), flavour or other characteristics | Marlboro Bright Leaf Platinum |
| Stock Keeping Unit (SKU) Each individual barcoded product including specific pack characteristics: pack size, whether the pack is price-marked, and, for FM but not RYO, whether standardised or branded | Marlboro Bright Leaf Platinum 10s multipack price marked |

# Description of tax calculations

Taxes were calculated at case level (SKU at a particular time point and geography) with the exception of ad valorem tax which was calculated at brand variant level because one tax level applied to all SKU within a brand variant.

## Value added tax (VAT)

VAT was a 20% tax (1) that was included in final retail prices, and was therefore calculated on the basis that the final retail price represented 120% of the product price without VAT, applicable each month

## Specific tobacco tax

Specific taxes were calculated as the applicable rate for each month divided by 1000 for FM (as the FM specific rate was per 1000 sticks) and divided by 2000 for RYO and MYO as the specific rate was per 1000 kilograms and 1 RYO stick was assumed to contain 0.5g tobacco) (2)

## Ad valorem tobacco tax

Ad valorem calculation was more complex. The HMRC calculate only one ad valorem rate for each brand variant (price marked packs did not incur a separate tax rate and the rate was calculated using the recommended retail price (RRP) of 20 stick packs and then applied proportionately to different pack sizes) (3, 4). However, some brands (pre standardised packaging) did not have a 20 stick variant.

We therefore estimated ad valorem tax in the following way:

**Sample restrictions:** we restricted the analysis to SKU that were distributed to 10% or more stores UK wide. For reliable estimates Nielsen recommends excluding more narrowly distributed SKU. HMRC base ad valorem on RRP but Nielsen supplies sales’ prices. Geographical price differences could mean that the price of a SKU widely distributed in one geography (but not overall) might not reflect the RRP. Thus our method of calculating ad valorem tax would be compromised so SKU that crossed the threshold for inclusion in a geography were excluded if their overall distribution did not reach the distribution threshold. This led to exclusions of a total of 0.2% or less of widely distributed FM sticks in most areas and 0.8% sticks in London and Scotland.

For the calculation of ad valorem tax rates only, we excluded 8 out of the 882 SKU that were widely distributed at some point over the data series because they were sold in 23 or 24 stick packs because of our reliance on 20 stick packs in the ad valorem tax calculations (see below). They were BAT brand variants: Pall Mall King Size Double Capsule, Rothman’s King Size Blue and Royals King Size Red. Thus 20 sticks were the largest pack size and theoretically should thus be the most expensive if they were being sold at the RRP.

**Identifying the SKU to use for calculations**: we identified which was the most expensive SKU each month for each brand variant and its pack size. If it was the 20 pack we used this price to calculate ad valorem. For packs containing less than 20 sticks, the ad valorem tax was calculated pro rata from the 20 stick equivalent. If there was no 20 pack then the highest pack size available was used to calculate ad valorem.

**Calculations:** A brand variant’s ad valorem was calculated by the pack price multiplied by the ad valorem rate (16.5%). This was then divided by the pack size to find the ad valorem per stick.

## Minimum excise tobacco tax (MET)

The excise tax refers to the sum of the ad valorem and specific tax elements. If the sum of these does not reach the government specified MET level, then the MET level is applicable. We thus calculated the total ad valorem and specific tax for each stick and compared that with the minimum excise tax level. If the total ad valorem and specific tax did not reach the threshold then the MET tax was taken instead of the total. There was only one variant where the MET was applicable: Chesterfield Superkings Red in June 2017 (the month following MET implementation). The UK wide pack price was £7.27 and geographical variations were between £7.11 (North East) and £7.36 (Scotland). The excise tax (specific plus ad valorem) was £5.34 and the minimum duty not to incur MET was £5.37; this was intended to set a floor for pack price at £7.35 (5).

# More detail on how time was modelled in GAMM and time trend estimation

## Addressing the non-linearity of time trends

Exploratory analysis showed that the trends of net revenue in time were non-linear (see figure S2). There appeared to be a non linear rise in prices in November 2017, a rise and than a plateau in FM mid price and value brands in 2015 to 2016, and a steep rise in sub value brands in early to mid 2017. RYO premium and mid price brands rose more steeply than RYO value brands. Thus although prices did appear to rise for all market segments the pattern and timing or rises varied somewhat, suggesting an interaction between price segment and time. There was little variation in geographical price patterns. However, it is not possible to infer the importance of price changes without statistical testing.

Figure B1 Raw real weighted* mean price per stick, by geography and market segment


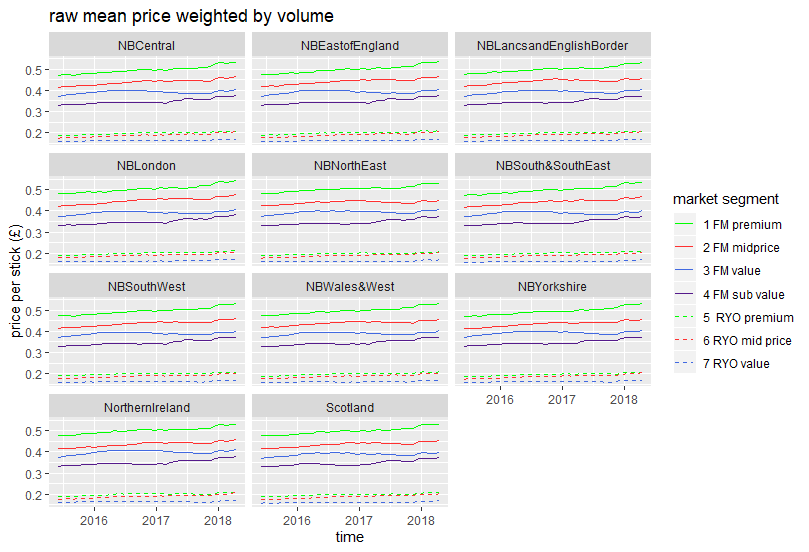


*weighted by sales volume of sticks (FM) or stick equivalents (RYO where one stick=0.5g tobacco)

Figure B2 Raw real weighted* mean net revenue per stick, by geography and market segment


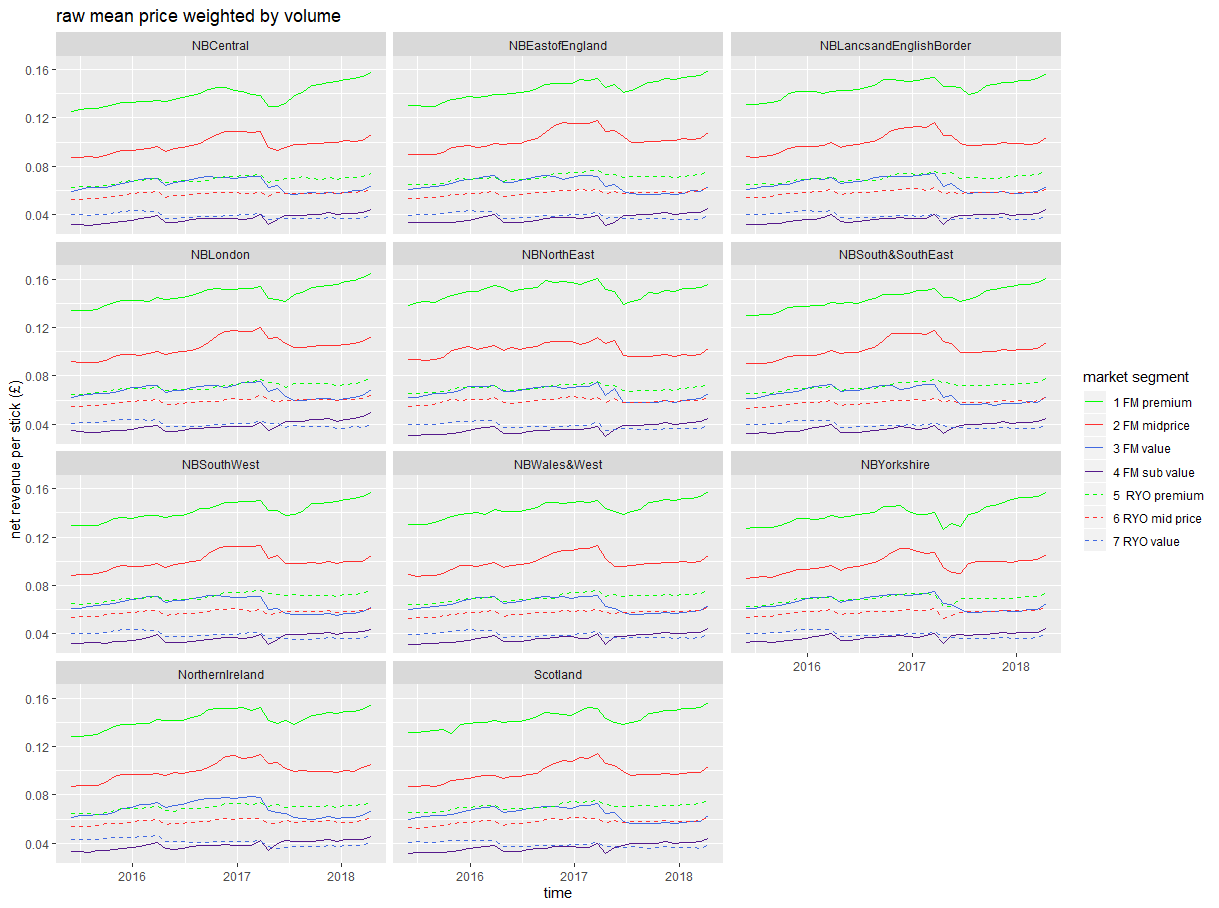


*weighted by sales volume of sticks (FM) or stick equivalents (RYO where one stick=0.5g tobacco)

## Addressing the lack of independence of monthly observations

Figure C shows the estimated auto-correlation function of residuals for observations of one SKU (as an example) pre and post adding an auto regressive process (AR1). Before the AR1 process was added the residuals are similar to their neighbours, shown by the high correlation ( > 0.4) shown on the y axis of observations which where between one and three months apart (lag 1 – 3 on x axis). After the AR1 process was included the correlation of the standardized residuals (i.e. after dividing by the square root of their covariance) is smaller.

Figure C Residuals for one SKU (a) without and (b) with an autoregressive process


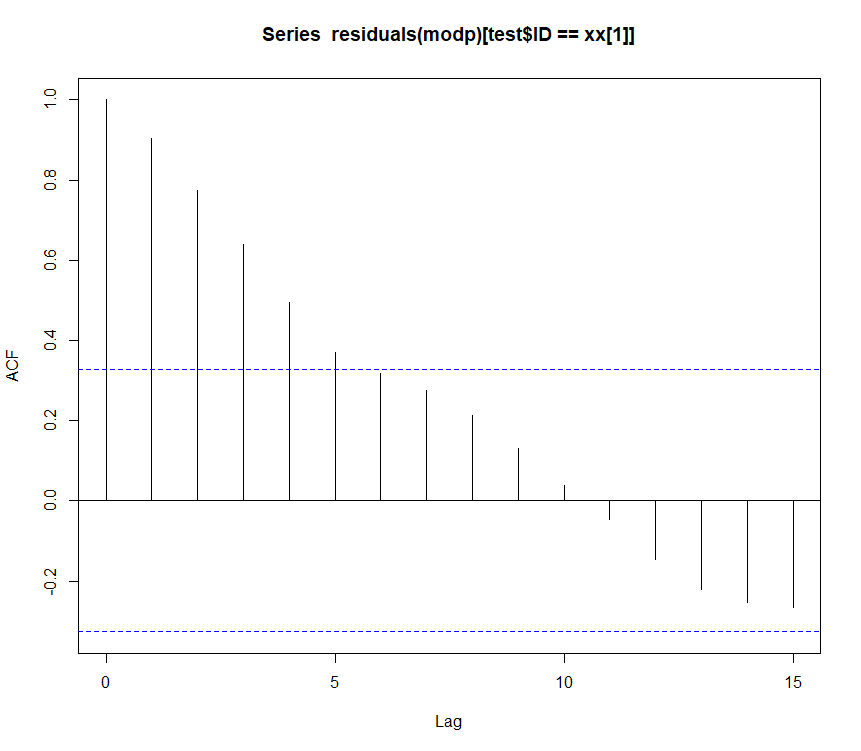

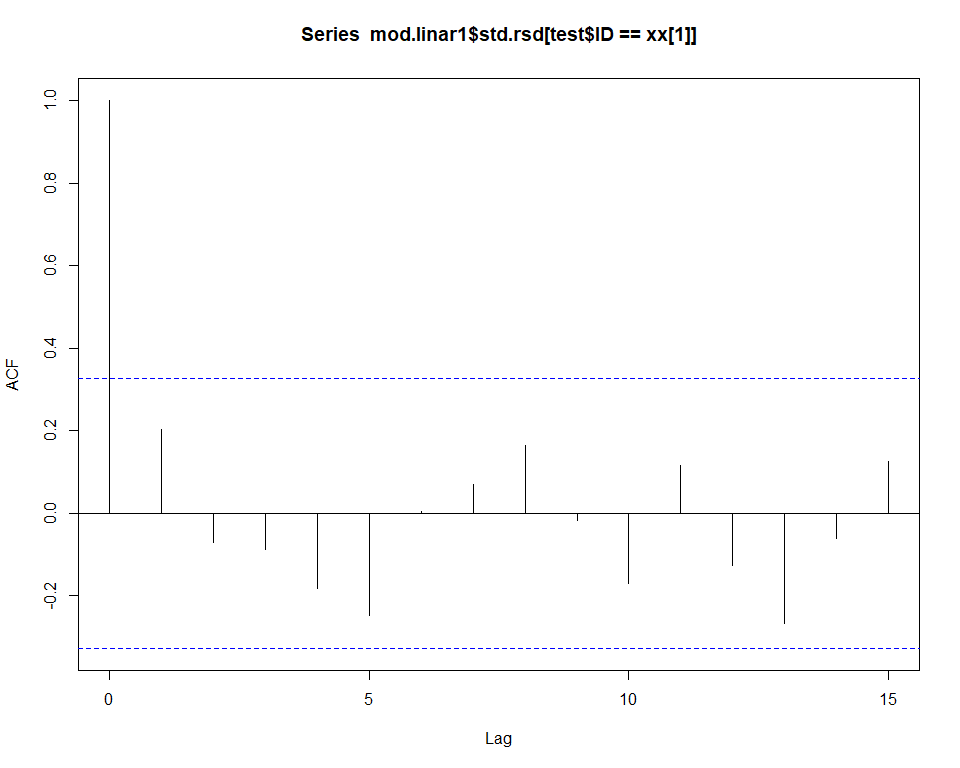


OTHER RESIDUAL DIAGNOSTIC PLOTS

Figure D shows that the normal assumption for residuals is reasonable (other distributions considered did not improve matters). The assumption of constant variance is also adequate because variability of residuals remains constant throughout the range of fitted values as we can see in the plot of residual vs the fitted values (Resids vs linear pred.).

Figure D Diagnostic residual plots of model fit (8) for the final AMMs for (a) price (b) net revenue

1. Price per stick


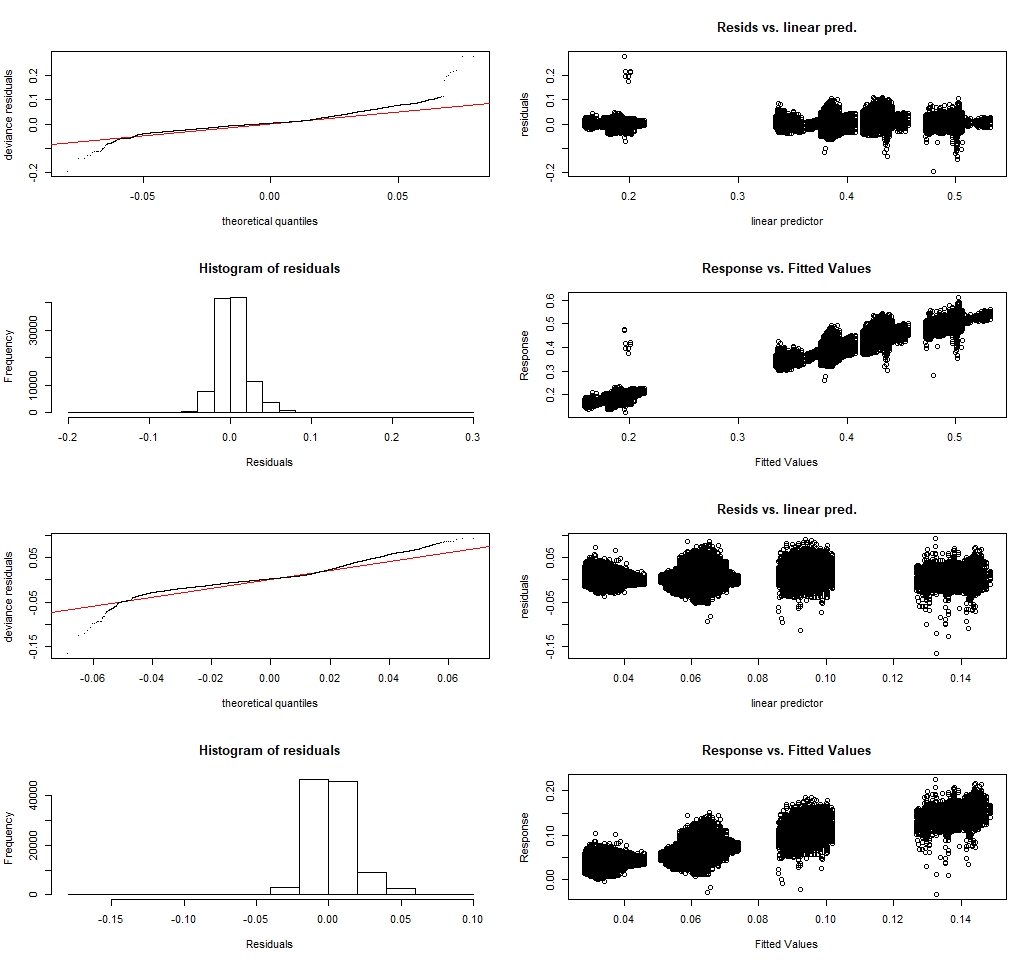


1. Net revenue per stick


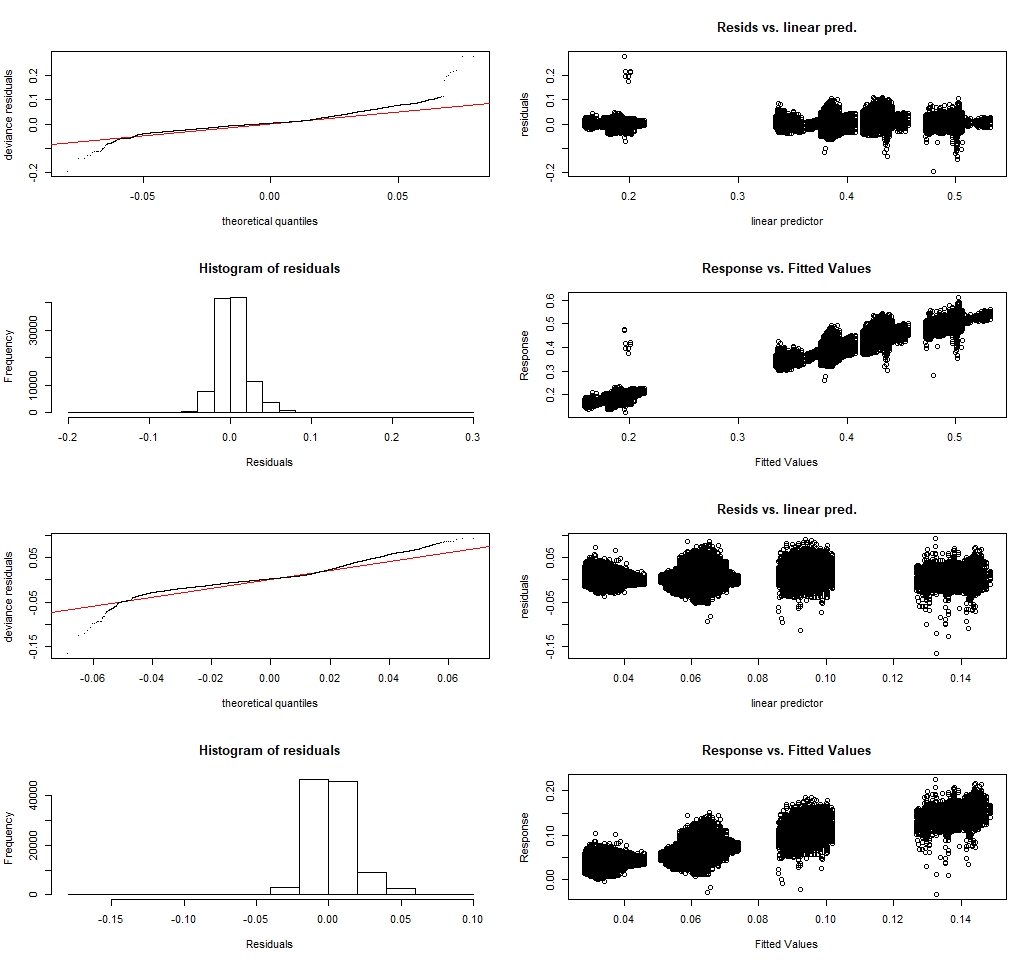


## Time trend estimation

For estimating the 95% credible intervals of the respective time trends (or any other statistic), we took samples from the multivariate normal posterior distribution of the model output coefficients (9). This was possible because the GAMM can be statistically be written down using a Bayesian representation (9-11). Then by using Bayes theorem, a multivariate normal posterior distribution of the model parameters was obtained. Hence, by sampling from the multivariate normal posterior distribution of the model coefficients the predictive distributions of any statistic of interest can be obtained; the lower and upper 95% quantiles constitute the Bayesian confidence intervals, also called credible intervals, for the quantity of interest. That is, for each sample of coefficients we estimated the required statistic. We then obtained the 0.975 and 0.025th quantiles (12, 13) from these statistics which are the 95% Bayesian confidence intervals. We used these type of intervals because in the context of GAMMs, the Bayesian credible intervals have been shown to have good coverage properties (11)). We displayed the median of the estimated means from the simulated samples in the graphs.

Calculation of **weighted average price**: Taking the mean of the estimated $\hat{pps_{i}}$’s over all SKUs *i* and by time and segment yielded estimates of time trends by segment. Weighting by the monthly market share of each SKU within segment yielded time trends based on the weighted average.

Calculation of the **gaps between weighted average prices over time** (Figure 3) were computed by adding an extra step of taking the difference between the time trends of the different product types and market segments.

For the **forest plots** (figure 4) we calculated, for each market segment, the mean monthly change in price (compared to the previous month) in the same months pre implementation of standardized packaging (June 2015 to March 2016) and post full implementation (June 2017 to March 2018). The confidence intervals were created using the above Bayesian procedure but using the mean monthly change in price for the two periods as statistic. The mean change and accompanying confidence intervals are then displayed in the forest plot.

To understand the implications of the changes over the period for industry net revenue we used the following procedures. First to understand net revenue changes pre and post full implementation of standardized packaging, an equivalent GAMM model was created substituting net revenue for price per stick. From this model and equivalent **forest plot** (Figure 4) to that for price per stick was displayed for net revenue per stick. Second to understand the impact of tobacco control policy changes on undershifting after tax changes, a line graph shows change in **net revenue after tax changes** (figure 5). Undershifting is shown for each market segment (a) after the March 2016 tax change (pre implementation of standardized packaging and MET) and (b) after the November 2017 tax change (post full implementation and implementation of the MET). Credible intervals are created as described above. These differences quantified the extent of undershifting of new taxation. The undershifting is known to reduce the impact of taxation on pricing.

# Regression results

Table B Regression model of price per stick dependent variable parameters

|  | Estimate | Std. Error | t value | p |  |
| --- | --- | --- | --- | --- | --- |
| (Intercept) | 0.492 | 0.001 | 563.915 | <.001 | *** |
|  |  |  |  |  |  |
| *Market segment* |  |  |  |  |  |
| Ref FM premium | 0 |  |  |  |  |
| FM midprice | -.065 | .001 | -81.646 | <.001 | *** |
| FM value | -.109 | .001 | -146.503 | <.001 | *** |
| FM subvalue | -.151 | .001 | -194.688 | <.001 | *** |
| RYO premium | -.296 | .001 | -226.517 | <.001 | *** |
| RYO midprice | -.311 | .001 | -284.298 | <.001 | *** |
| RYO value | -.330 | .001 | -320.411 | <.001 | *** |
| no segment | -.304 | .001 | -214.256 | <.001 | *** |
|  |  |  |  |  |  |
| *Geography* |  |  |  |  |  |
| Ref Central England | 0 |  |  |  |  |
| East of England | .002 | .001 | 2.561 | .010 | * |
| Lancs and English Border | .002 | .001 | 2.302 | .021 | * |
| London | .005 | .001 | 6.277 | <.001 | *** |
| North East | .003 | .001 | 3.436 | .001 | *** |
| South & South East | .003 | .001 | 3.426 | .001 | *** |
| South West | .001 | .001 | .625 | .532 |  |
| Wales &West | .000 | .001 | .047 | .963 |  |
| Yorkshire | .001 | .001 | 1.079 | .281 |  |
| Northern Ireland | .002 | .001 | 2.249 | .024 | * |
| Scotland | .001 | .001 | .766 | .444 |  |
|  |  |  |  |  |  |
| *Smooth terms (interaction of segment*time)* | edf^1^ | Ref.df^2^ | F | p-value |  |
| FM premium | 18.94 | 19.00 | 282.03 | <.001 | *** |
| FM midprice | 18.94 | 19.00 | 246.66 | <.001 | *** |
| FM value | 18.97 | 19.00 | 344.07 | <.001 | *** |
| FM subvalue | 18.92 | 19.00 | 256.86 | <.001 | *** |
| RYO premium | 17.71 | 18.82 | 17.29 | <.001 | *** |
| RYO midprice | 18.17 | 18.92 | 22.62 | <.001 | *** |
| RYO value | 18.19 | 18.92 | 15.62 | <.001 | *** |
| no segment | 15.70 | 17.93 | 7.08 | <.001 | *** |
| R-sq.(adj) = 0.97 Deviance explained = 96.4%  fREML^3^ = 436670 Scale est^4^. = .00032239 n = 107571 | | | | | |

*** p<.001; **p<.01; *p<.05; ^1^ed =expected degrees of freedom; ^2^Ref.df=reference degrees of freedom; ^3^fREML is the score of the restriced maximum likelihood; ^4^scale est is the estimate of the variance of the errors in the model

Table C Comparison of price per stick model coefficients of (1) a linear model, (2) a linear model +AR1 and (3) an additive mixed model (AMM) (which also includes an AR1)

|  | Linear^1^ | | Linear+AR1 | | AMM | |
| --- | --- | --- | --- | --- | --- | --- |
|  | est | p | est | p | est | p |
| (Intercept) | 0.47420 | *** | 0.47040 | *** | 0.49200 | *** |
|  |  |  |  |  |  |  |
| *Time* | 0.00139 | *** | 0.00138 | *** | NA^2^ |  |
|  |  |  |  |  |  |  |
| *Market segment* |  |  |  |  |  |  |
| Ref FM premium | 0 |  | 0 |  | 0 |  |
| FM midprice | -0.05985 | *** | -0.05776 | *** | -0.06524 | *** |
| FM value | -0.09551 | *** | -0.09641 | *** | -0.10910 | *** |
| FM subvalue | -0.14720 | *** | -0.14080 | *** | -0.15140 | *** |
| RYO premium | -0.28680 | *** | -0.28420 | *** | -0.29640 | *** |
| RYO midprice | -0.29950 | *** | -0.29850 | *** | -0.31120 | *** |
| RYO value | -0.31570 | *** | -0.31290 | *** | -0.33020 | *** |
| no segment | -0.28860 | *** | -0.29090 | *** | -0.30370 | *** |
|  |  |  |  |  |  |  |
| *Geography* |  |  |  |  |  |  |
| Ref Central England | 0 |  | 0 |  | 0 |  |
| East of England | 0.00208 | *** | 0.00203 | * | 0.00205 | * |
| Lancs and English Border | 0.00193 | *** | 0.00180 | * | 0.00186 | * |
| London | 0.00407 | *** | 0.00502 | *** | 0.00507 | *** |
| North East | 0.00320 | *** | 0.00280 | ** | 0.00287 | *** |
| South & South East | 0.00238 | *** | 0.00278 | ** | 0.00278 | *** |
| South West | 0.00088 | *** | 0.00049 |  | 0.00051 |  |
| Wales &West | 0.00090 | *** | -0.00001 |  | 0.00004 |  |
| Yorkshire | 0.00088 | *** | 0.00084 |  | 0.00086 |  |
| Northern Ireland | 0.00334 | *** | 0.00185 | * | 0.00188 | * |
| Scotland | 0.00060 | * | 0.00055 |  | 0.00062 |  |
|  |  |  |  |  |  |  |
|  |  |  |  |  |  |  |
| *Interaction of segment*time* |  |  |  |  |  |  |
| FM premium | 0 |  | 0 |  | NA | *** |
| FM midprice | -0.00012 | *** | -0.00041 | *** | NA | *** |
| FM value | -0.00091 | *** | -0.00077 | *** | NA | *** |
| FM subvalue | -0.00041 | *** | -0.00066 | *** | NA | *** |
| RYO premium | -0.00078 | *** | -0.00077 | *** | NA | *** |
| RYO midprice | -0.00087 | *** | -0.00081 | *** | NA | *** |
| RYO value | -0.00119 | *** | -0.00109 | *** | NA | *** |
| no segment | -0.00131 | *** | -0.00082 | *** | NA | *** |

^1^ There was one more case in the linear model (n=107572) than the models including an AR1 (n=107571): the coding of the variable necessary for marking the start of a sequence of monthly SKU observations created one missing value at the start of the data set.

^2^NA: not available as entered as a smooth term

Table D Regression model of net revenue dependent variable parameters

|  | Estimate | Std. Error | t value | p |  |
| --- | --- | --- | --- | --- | --- |
| (Intercept) | .136 | .001 | 180.568 | <.001 | *** |
|  |  |  |  |  |  |
| *Market segment* |  |  |  |  |  |
| Ref FM premium | 0 |  |  |  |  |
| FM midprice | -.045 | .001 | -65.158 | <.001 | *** |
| FM value | -.074 | .001 | -115.035 | <.001 | *** |
| FM subvalue | -.103 | .001 | -153.349 | <.001 | *** |
| RYO premium | -.069 | .001 | -61.628 | <.001 | *** |
| RYO midprice | -.082 | .001 | -86.826 | <.001 | *** |
| RYO value | -.098 | .001 | -110.138 | <.001 | *** |
| no segment | -.079 | .001 | -65.089 | <.001 | *** |
|  |  |  |  |  |  |
| *Geography* |  |  |  |  |  |
| Ref Central England | 0 |  |  |  |  |
| East of England | .002 | .001 | 2.774 | .006 | ** |
| Lancs and English Border | .002 | .001 | 2.482 | .013 | * |
| London | .004 | .001 | 5.805 | .000 | *** |
| North East | .002 | .001 | 3.438 | .001 | *** |
| South & South East | .002 | .001 | 3.283 | .001 | ** |
| South West | .001 | .001 | 0.800 | .423 |  |
| Wales &West | .000 | .001 | 0.326 | .745 |  |
| Yorkshire | .001 | .001 | 1.238 | .216 |  |
| Northern Ireland | .002 | .001 | 2.306 | .021 | * |
| Scotland | .001 | .001 | 0.813 | .416 |  |
|  |  |  |  |  |  |
| *Smooth terms (interaction of segment*time)* | edf^1^ | Ref.df^2^ | F | p-value |  |
| FM premium | 18.84 | 19.00 | 223.369 | <.001 | *** |
| FM midprice | 18.94 | 19.00 | 310.877 | <.001 | *** |
| FM value | 18.95 | 19.00 | 338.634 | <.001 | *** |
| FM subvalue | 18.94 | 19.00 | 203.132 | <.001 | *** |
| RYO premium | 18.27 | 18.94 | 21.928 | <.001 | *** |
| RYO midprice | 18.33 | 18.95 | 25.341 | <.001 | *** |
| RYO value | 18.54 | 18.97 | 39.964 | <.001 | *** |
| no segment | 17.34 | 18.70 | 7.709 | <.001 | *** |
| R-sq.(adj) = .815 Deviance explained = 80.9%  fREML^3^ =-453800 Scale est^4^. =.00023878 n = 107571 | | | | | |

*** p<.001; **p<.01; *p<.05; ^1^ed =expected degrees of freedom; ^2^Ref.df=reference degrees of freedom; ^3^fREML is the score of the restriced maximum likelihood; ^4^scale est is the estimate of the variance of the errors in the mode

There were significant differences in price per stick across the time series by geography and market segment (see table B). Highest prices were found in London, Northern, Eastern, and Southern England, and Northern Ireland. As expected, premium FM cigarettes were significantly higher priced than all other market segments. Similar patterns were found for net revenue (see table D).

Table C shows similar coefficients for a linear model, a linear model with an AR1 and our final price per stick AMM. Including an AR1 reduces the significance of the differences between some geographical areas.

# Modelled stick prices overall by tobacco type and market segment

Table E Price per stick over time overall and by tobacco type

|  | total |  |  | FM |  |  | RYO |  |
| --- | --- | --- | --- | --- | --- | --- | --- | --- |
|  | price per stick (£) (95%CI) | difference (£) from previous month |  | price per stick (£) (95%CI) | difference (£) from previous month |  | price per stick (£) (95%CI) | difference (£) from previous month |
| May-15 | .3313 (.3308 to .3319) |  |  | .3964 (.3959 to .3969) |  |  | .1755 (.1742 to .1768) |  |
| Jun-15 | .3315 (.3310 to .3321) | .000 |  | .3966 (.3961 to .3971) | .000 |  | .1758 (.1745 to .1770) | .000 |
| Jul-15 | .3315 (.3310 to .3321) | .000 |  | .3962 (.3957 to .3967) | .000 |  | .1760 (.1747 to .1772) | .000 |
| Aug-15 | .3314 (.3309 to .3320) | .000 |  | .3957 (.3952 to .3962) | -.001 |  | .1762 (.1749 to .1775) | .000 |
| Sep-15 | .3315 (.3310 to .3321) | .000 |  | .3963 (.3958 to .3968) | .001 |  | .1769 (.1756 to .1781) | .001 |
| Oct-15 | .3327 (.3322 to .3333) | .001 |  | .3973 (.3969 to .3978) | .001 |  | .1780 (.1768 to .1793) | .001 |
| Nov-15 | .3337 (.3332 to .3343) | .001 |  | .3989 (.3985 to .3994) | .002 |  | .1790 (.1778 to .1802) | .001 |
| Dec-15 | .3350 (.3345 to .3356) | .001 |  | .3998 (.3993 to .4003) | .001 |  | .1797 (.1785 to .1809) | .001 |
| Jan-16 | .3315 (.3310 to .3321) | -.004 |  | .3991 (.3987 to .3996) | -.001 |  | .1799 (.1787 to .1811) | .000 |
| Feb-16 | .3311 (.3306 to .3316) | .000 |  | .3992 (.3987 to .3997) | .000 |  | .1804 (.1793 to .1816) | .001 |
| Mar-16 | .3318 (.3313 to .3323) | .001 |  | .3996 (.3991 to .4001) | .000 |  | .1813 (.1801 to .1825) | .001 |
| Apr-16 | .3316 (.3311 to .3321) | .000 |  | .3999 (.3995 to .4004) | .000 |  | .1822 (.1809 to .1833) | .001 |
| May-16 | .3325 (.3320 to .3330) | .001 |  | .3996 (.3992 to .4001) | .000 |  | .1827 (.1814 to .1839) | .001 |
| Jun-16 | .3322 (.3317 to .3327) | .000 |  | .3990 (.3985 to .3995) | -.001 |  | .1829 (.1817 to .1842) | .000 |
| Jul-16 | .3319 (.3314 to .3325) | .000 |  | .3987 (.3982 to .3992) | .000 |  | .1832 (.1820 to .1844) | .000 |
| Aug-16 | .3315 (.3310 to .3320) | .000 |  | .3986 (.3982 to .3991) | .000 |  | .1832 (.1820 to .1845) | .000 |
| Sep-16 | .3302 (.3297 to .3308) | -.001 |  | .3982 (.3977 to .3986) | .000 |  | .1829 (.1817 to .1841) | .000 |
| Oct-16 | .3307 (.3302 to .3313) | .000 |  | .3973 (.3968 to .3978) | -.001 |  | .1826 (.1814 to .1837) | .000 |
| Nov-16 | .3296 (.3290 to .3301) | -.001 |  | .3960 (.3955 to .3965) | -.001 |  | .1829 (.1817 to .1840) | .000 |
| Dec-16 | .3307 (.3302 to .3313) | .001 |  | .3963 (.3958 to .3968) | .000 |  | .1830 (.1818 to .1841) | .000 |
| Jan-17 | .3272 (.3266 to .3277) | -.004 |  | .3967 (.3962 to .3972) | .000 |  | .1827 (.1815 to .1839) | .000 |
| Feb-17 | .3281 (.3276 to .3287) | .001 |  | .3976 (.3971 to .3981) | .001 |  | .1834 (.1821 to .1845) | .001 |
| Mar-17 | .3294 (.3288 to .3300) | .001 |  | .3990 (.3985 to .3995) | .001 |  | .1841 (.1827 to .1853) | .001 |
| Apr-17 | .3305 (.3300 to .3311) | .001 |  | .3995 (.3990 to .4000) | .001 |  | .1849 (.1836 to .1861) | .001 |
| May-17 | .3311 (.3305 to .3316) | .001 |  | .4004 (.3999 to .4010) | .001 |  | .1859 (.1847 to .1871) | .001 |
| Jun-17 | .3299 (.3293 to .3305) | -.001 |  | .3997 (.3991 to .4002) | -.001 |  | .1857 (.1844 to .1869) | .000 |
| Jul-17 | .3299 (.3293 to .3305) | .000 |  | .3994 (.3988 to .4000) | .000 |  | .1852 (.1838 to .1864) | -.001 |
| Aug-17 | .3284 (.3277 to .3290) | -.002 |  | .3990 (.3984 to .3997) | .000 |  | .1845 (.1830 to .1857) | -.001 |
| Sep-17 | .3262 (.3256 to .3268) | -.002 |  | .3975 (.3968 to .3981) | -.002 |  | .1835 (.1821 to .1848) | -.001 |
| Oct-17 | .3255 (.3248 to .3261) | -.001 |  | .3957 (.3950 to .3964) | -.002 |  | .1828 (.1814 to .1841) | -.001 |
| Nov-17 | .3280 (.3273 to .3286) | .003 |  | .3996 (.3988 to .4002) | .004 |  | .1845 (.1831 to .1859) | .002 |
| Dec-17 | .3348 (.3342 to .3355) | .007 |  | .4075 (.4068 to .4082) | .008 |  | .1881 (.1866 to .1895) | .004 |
| Jan-18 | .3343 (.3336 to .3350) | -.001 |  | .4098 (.4091 to .4106) | .002 |  | .1893 (.1877 to .1907) | .001 |
| Feb-18 | .3326 (.3318 to .3333) | -.002 |  | .4085 (.4078 to .4094) | -.001 |  | .1890 (.1874 to .1905) | .000 |
| Mar-18 | .3328 (.3321 to .3335) | .000 |  | .4094 (.4087 to .4102) | .001 |  | .1896 (.1880 to .1910) | .001 |
| Apr-18 | .3353 (.3346 to .3361) | .003 |  | .4123 (.4115 to .4131) | .003 |  | .1912 (.1895 to .1927) | .002 |

Table F price per stick over time by FM market segment

|  | FM premium |  | FM midprice |  | FM value |  | FM subvalue |  |
| --- | --- | --- | --- | --- | --- | --- | --- | --- |
|  | price per stick (£) (95%CI) | difference (£) from previous month | price per stick (£) (95%CI) | difference (£) from previous month | price per stick (£) (95%CI) | difference (£) from previous month | price per stick (£) (95%CI) | difference (£) from previous month |
| May-15 | .4748 (.4730 to .4763) |  | .4163 (.4152 to .4173) |  | .3769 (.3762 to .3777) |  | .3355 (.3345 to .3365) |  |
| Jun-15 | .4753 (.4736 to .4769) | .001 | .4167 (.4157 to .4177) | .000 | .3775 (.3768 to .3782) | .001 | .3359 (.3349 to .3368) | .000 |
| Jul-15 | .4755 (.4739 to .4772) | .000 | .4168 (.4158 to .4178) | .000 | .3780 (.3773 to .3787) | .001 | .3363 (.3353 to .3372) | .000 |
| Aug-15 | .4758 (.4741 to .4774) | .000 | .4167 (.4158 to .4177) | .000 | .3785 (.3778 to .3792) | .000 | .3367 (.3358 to .3376) | .000 |
| Sep-15 | .4777 (.4761 to .4794) | .002 | .4177 (.4168 to .4187) | .001 | .3792 (.3785 to .3798) | .001 | .3371 (.3362 to .3380) | .000 |
| Oct-15 | .4814 (.4798 to .4831) | .004 | .4202 (.4192 to .4211) | .002 | .3802 (.3796 to .3809) | .001 | .3375 (.3367 to .3384) | .000 |
| Nov-15 | .4843 (.4828 to .4861) | .003 | .4223 (.4214 to .4232) | .002 | .3818 (.3811 to .3824) | .002 | .3384 (.3375 to .3392) | .001 |
| Dec-15 | .4853 (.4837 to .4871) | .001 | .4234 (.4225 to .4243) | .001 | .3833 (.3827 to .3840) | .002 | .3394 (.3386 to .3403) | .001 |
| Jan-16 | .4849 (.4834 to .4867) | .000 | .4237 (.4227 to .4246) | .000 | .3842 (.3835 to .3848) | .001 | .3398 (.3389 to .3406) | .000 |
| Feb-16 | .4854 (.4840 to .4871) | .001 | .4240 (.4231 to .4249) | .000 | .3844 (.3837 to .3851) | .000 | .3396 (.3388 to .3405) | .000 |
| Mar-16 | .4893 (.4879 to .4910) | .004 | .4253 (.4244 to .4262) | .001 | .3846 (.3839 to .3853) | .000 | .3395 (.3386 to .3403) | .000 |
| Apr-16 | .4938 (.4924 to .4954) | .005 | .4268 (.4259 to .4277) | .001 | .3847 (.3840 to .3854) | .000 | .3393 (.3384 to .3401) | .000 |
| May-16 | .4951 (.4935 to .4966) | .001 | .4275 (.4265 to .4284) | .001 | .3845 (.3838 to .3852) | .000 | .3386 (.3378 to .3395) | -.001 |
| Jun-16 | .4951 (.4935 to .4967) | .000 | .4278 (.4269 to .4287) | .000 | .3845 (.3838 to .3852) | .000 | .3381 (.3372 to .3390) | -.001 |
| Jul-16 | .4966 (.4952 to .4983) | .002 | .4286 (.4276 to .4295) | .001 | .3849 (.3842 to .3856) | .000 | .3381 (.3372 to .3390) | .000 |
| Aug-16 | .4994 (.4978 to .5010) | .003 | .4295 (.4285 to .4304) | .001 | .3853 (.3847 to .3861) | .000 | .3382 (.3373 to .3391) | .000 |
| Sep-16 | .5020 (.5006 to .5036) | .003 | .4304 (.4294 to .4313) | .001 | .3852 (.3845 to .3859) | .000 | .3379 (.3370 to .3388) | .000 |
| Oct-16 | .5025 (.5009 to .5040) | .000 | .4312 (.4302 to .4321) | .001 | .3840 (.3833 to .3847) | -.001 | .3379 (.3370 to .3388) | .000 |
| Nov-16 | .5010 (.4995 to .5026) | -.001 | .4318 (.4308 to .4328) | .001 | .3824 (.3817 to .3832) | -.002 | .3381 (.3372 to .3390) | .000 |
| Dec-16 | .5007 (.4993 to .5023) | .000 | .4324 (.4314 to .4334) | .001 | .3826 (.3818 to .3833) | .000 | .3383 (.3374 to .3392) | .000 |
| Jan-17 | .5020 (.5005 to .5036) | .001 | .4336 (.4325 to .4346) | .001 | .3839 (.3832 to .3847) | .001 | .3388 (.3378 to .3396) | .000 |
| Feb-17 | .5027 (.5013 to .5043) | .001 | .4357 (.4347 to .4368) | .002 | .3843 (.3836 to .3850) | .000 | .3404 (.3394 to .3412) | .002 |
| Mar-17 | .5029 (.5015 to .5045) | .000 | .4378 (.4368 to .4389) | .002 | .3846 (.3839 to .3854) | .000 | .3433 (.3423 to .3443) | .003 |
| Apr-17 | .5032 (.5018 to .5048) | .000 | .4383 (.4373 to .4394) | .001 | .3870 (.3863 to .3878) | .002 | .3475 (.3464 to .3485) | .004 |
| May-17 | .5020 (.5005 to .5036) | -.001 | .4373 (.4362 to .4384) | -.001 | .3896 (.3888 to .3903) | .003 | .3517 (.3506 to .3527) | .004 |
| Jun-17 | .4988 (.4973 to .5005) | -.003 | .4355 (.4345 to .4367) | -.002 | .3898 (.3890 to .3906) | .000 | .3548 (.3537 to .3559) | .003 |
| Jul-17 | .4985 (.4970 to .5001) | .000 | .4347 (.4336 to .4359) | -.001 | .3892 (.3883 to .3901) | -.001 | .3564 (.3552 to .3575) | .002 |
| Aug-17 | .5030 (.5014 to .5048) | .005 | .4351 (.4339 to .4364) | .000 | .3889 (.3880 to .3898) | .000 | .3562 (.3550 to .3574) | .000 |
| Sep-17 | .5059 (.5041 to .5077) | .003 | .4338 (.4324 to .4351) | -.001 | .3871 (.3861 to .3880) | -.002 | .3543 (.3531 to .3555) | -.002 |
| Oct-17 | .5058 (.5040 to .5076) | .000 | .4318 (.4304 to .4332) | -.002 | .3852 (.3841 to .3862) | -.002 | .3531 (.3518 to .3543) | -.001 |
| Nov-17 | .5110 (.5093 to .5129) | .005 | .4362 (.4348 to .4376) | .004 | .3892 (.3881 to .3902) | .004 | .3573 (.3560 to .3586) | .004 |
| Dec-17 | .5206 (.5188 to .5224) | .010 | .4449 (.4435 to .4463) | .009 | .3972 (.3961 to .3983) | .008 | .3650 (.3637 to .3664) | .008 |
| Jan-18 | .5251 (.5233 to .5270) | .005 | .4481 (.4466 to .4496) | .003 | .4001 (.3990 to .4012) | .003 | .3682 (.3669 to .3696) | .003 |
| Feb-18 | .5250 (.5231 to .5269) | .000 | .4469 (.4454 to .4485) | -.001 | .3990 (.3980 to .4001) | -.001 | .3676 (.3662 to .3690) | -.001 |
| Mar-18 | .5260 (.5242 to .5281) | .001 | .4485 (.4470 to .4501) | .002 | .4003 (.3992 to .4014) | .001 | .3682 (.3668 to .3697) | .001 |
| Apr-18 | .5289 (.5270 to .5310) | .003 | .4528 (.4512 to .4544) | .004 | .4040 (.4028 to .4052) | .004 | .3704 (.3690 to .3720) | .002 |

Table G price per stick over time by RYO market segment

|  | RYO premium |  |  | RYO midprice |  |  | RYO value |  |
| --- | --- | --- | --- | --- | --- | --- | --- | --- |
|  | price per stick (£) (95%CI) | difference (£) from previous month |  | price per stick (£) (95%CI) | difference (£) from previous month |  | price per stick (£) (95%CI) | difference (£) from previous month |
| May-15 | .1887 (.1862 to .1914) |  |  | .1743 (.1721 to .1762) |  |  | .1600 (.1580 to .1619) |  |
| Jun-15 | .1889 (.1865 to .1917) | .000 |  | .1747 (.1725 to .1766) | .000 |  | .1602 (.1582 to .1621) | .000 |
| Jul-15 | .1890 (.1866 to .1917) | .000 |  | .1751 (.1729 to .1771) | .000 |  | .1603 (.1584 to .1622) | .000 |
| Aug-15 | .1891 (.1866 to .1918) | .000 |  | .1756 (.1734 to .1776) | .000 |  | .1605 (.1586 to .1623) | .000 |
| Sep-15 | .1899 (.1875 to .1926) | .001 |  | .1763 (.1743 to .1783) | .001 |  | .1610 (.1591 to .1628) | .000 |
| Oct-15 | .1916 (.1891 to .1944) | .002 |  | .1774 (.1754 to .1794) | .001 |  | .1617 (.1599 to .1636) | .001 |
| Nov-15 | .1931 (.1906 to .1958) | .001 |  | .1783 (.1765 to .1803) | .001 |  | .1625 (.1607 to .1643) | .001 |
| Dec-15 | .1938 (.1912 to .1965) | .001 |  | .1790 (.1771 to .1810) | .001 |  | .1629 (.1611 to .1648) | .000 |
| Jan-16 | .1941 (.1915 to .1967) | .000 |  | .1796 (.1777 to .1815) | .001 |  | .1629 (.1612 to .1648) | .000 |
| Feb-16 | .1947 (.1921 to .1973) | .001 |  | .1804 (.1785 to .1823) | .001 |  | .1628 (.1611 to .1646) | .000 |
| Mar-16 | .1961 (.1936 to .1988) | .001 |  | .1814 (.1795 to .1833) | .001 |  | .1628 (.1611 to .1646) | .000 |
| Apr-16 | .1977 (.1953 to .2005) | .002 |  | .1825 (.1807 to .1844) | .001 |  | .1628 (.1611 to .1646) | .000 |
| May-16 | .1985 (.1960 to .2013) | .001 |  | .1831 (.1813 to .1850) | .001 |  | .1628 (.1611 to .1645) | .000 |
| Jun-16 | .1987 (.1963 to .2014) | .000 |  | .1837 (.1818 to .1855) | .001 |  | .1628 (.1611 to .1646) | .000 |
| Jul-16 | .1987 (.1962 to .2014) | .000 |  | .1842 (.1824 to .1861) | .001 |  | .1629 (.1613 to .1647) | .000 |
| Aug-16 | .1987 (.1961 to .2013) | .000 |  | .1844 (.1826 to .1863) | .000 |  | .1630 (.1613 to .1647) | .000 |
| Sep-16 | .1987 (.1961 to .2014) | .000 |  | .1842 (.1824 to .1861) | .000 |  | .1628 (.1610 to .1645) | .000 |
| Oct-16 | .1990 (.1965 to .2019) | .000 |  | .1839 (.1821 to .1858) | .000 |  | .1626 (.1609 to .1643) | .000 |
| Nov-16 | .1998 (.1974 to .2026) | .001 |  | .1839 (.1821 to .1859) | .000 |  | .1626 (.1609 to .1642) | .000 |
| Dec-16 | .2004 (.1979 to .2031) | .001 |  | .1840 (.1822 to .1859) | .000 |  | .1626 (.1610 to .1642) | .000 |
| Jan-17 | .2008 (.1982 to .2034) | .000 |  | .1841 (.1822 to .1859) | .000 |  | .1628 (.1612 to .1644) | .000 |
| Feb-17 | .2016 (.1990 to .2043) | .001 |  | .1841 (.1822 to .1860) | .000 |  | .1634 (.1618 to .1650) | .001 |
| Mar-17 | .2025 (.1999 to .2051) | .001 |  | .1846 (.1827 to .1866) | .001 |  | .1644 (.1628 to .1660) | .001 |
| Apr-17 | .2028 (.2001 to .2053) | .000 |  | .1862 (.1841 to .1882) | .002 |  | .1658 (.1640 to .1674) | .001 |
| May-17 | .2025 (.1998 to .2051) | .000 |  | .1875 (.1854 to .1895) | .001 |  | .1667 (.1650 to .1686) | .001 |
| Jun-17 | .2022 (.1993 to .2047) | .000 |  | .1878 (.1856 to .1897) | .000 |  | .1667 (.1649 to .1685) | .000 |
| Jul-17 | .2020 (.1990 to .2046) | .000 |  | .1875 (.1852 to .1895) | .000 |  | .1661 (.1642 to .1680) | -.001 |
| Aug-17 | .2018 (.1988 to .2044) | .000 |  | .1875 (.1851 to .1895) | .000 |  | .1655 (.1635 to .1675) | -.001 |
| Sep-17 | .2010 (.1979 to .2038) | -.001 |  | .1868 (.1844 to .1890) | -.001 |  | .1646 (.1625 to .1667) | -.001 |
| Oct-17 | .2002 (.1972 to .2031) | -.001 |  | .1861 (.1837 to .1883) | -.001 |  | .1641 (.1620 to .1662) | -.001 |
| Nov-17 | .2019 (.1989 to .2050) | .002 |  | .1881 (.1856 to .1903) | .002 |  | .1660 (.1640 to .1682) | .002 |
| Dec-17 | .2055 (.2022 to .2086) | .004 |  | .1917 (.1891 to .1941) | .004 |  | .1695 (.1674 to .1718) | .003 |
| Jan-18 | .2073 (.2040 to .2105) | .002 |  | .1932 (.1906 to .1957) | .001 |  | .1708 (.1685 to .1730) | .001 |
| Feb-18 | .2076 (.2043 to .2109) | .000 |  | .1929 (.1902 to .1956) | .000 |  | .1702 (.1679 to .1725) | -.001 |
| Mar-18 | .2086 (.2051 to .2119) | .001 |  | .1935 (.1909 to .1963) | .001 |  | .1708 (.1686 to .1731) | .001 |
| Apr-18 | .2103 (.2068 to .2138) | .002 |  | .1952 (.1925 to .1980) | .002 |  | .1726 (.1702 to .1749) | .002 |

Table H Average monthly rise in price per stick (£) by market segment pre and post full implementation of standardised packaging and MET

|  | Slope pre (95%CI)^1^ | Slope post (95%CI)^2^ |
| --- | --- | --- |
| FM |  |  |
| Premium | .0019 (.0018 to .0020) | .0030 (.0029 to .0032) |
| Midprice | .0010 (.0009 to .0011) | .0017 (.0016 to .0019) |
| Value | .0007 (.0007 to .0008) | .0014 (.0013 to .0015) |
| Subvalue | .0003 (.0003 to .0004) | .0016 (.0014 to .0017) |
|  |  |  |
| RYO |  |  |
| Premium | .0009 (.0007 to .0011) | .0008 (.0006 to .0011) |
| Midprice | .0008 (.0007 to .0009) | .0007 (.0005 to .0010) |
| Value | .0003 (.0001 to .000390) | .0006 (.000393 to .0008) |

^1^Slope pre=June 2015 to March 2016: pre implementation of the MET and standardised packaging

^2^Slope post=June 2016 to March 2017: post implementation of the MET and post full implementation of standardised packaging

# R code for GAMM fitting and trend estimation

library(MASS)

library(mgcv)

library(ggplot2)

load("dat.RData") # read in prepared Nielsen data

set.seed(8)

levels(dat$seg8pre)<-c("1 FM premium", "2 FM midprice", "3 FM value", "4 FM sub value", "5 RYO premium", "6 RYO mid price", "7 RYO value", "8 no segment")

dat$tax<-dat$ppsvc-dat$netpricevc

dat$time<-as.numeric(dat$monthf)

dat<-dat[!is.na(dat$tax),] # this removes observations which didn't have distr. >9 in total coverage so tax couldnt be calculated'

dat$region<-factor(dat$region)

# set up month variable

month<- as.numeric(dat$monthf)

indi <- month>12 &month<25

monthnew<-month

monthnew[indi] <- month[indi] - 12

indi <- month> 24

monthnew[indi] <- month[indi] - 24

dat$month<-monthnew

dat<-dat[dat$region != "TotalCoverage",] # only use regional data

test<-dat[dat$dtb>9 ,] # only use data with distribution greater 9%

# set up index of 0 and 1 with 1 if the time series of monthly prices starts for SKU and

# region or if it restarts after some months with missing values

# this is needed for the AR1 in the model

sortindex<- order(test$ID, test$time)

test<- test[sortindex,]

test$timel1 <- c(NA,test$time[-length(test$time)])

test$arstart<-test$time-test$timel1 != 1

test$region <-factor(test$region)

levels(test$typef)

levels(test$typef) <- c("FM", "RYO", "RYO") # combine last two levels (i.e. treat RYO and MYO as the same)

# Model for price per stick

# first estimate numerically the parameter phi for the AR1 numerically by minimising aic

# using a for loop

aic <- reml <- rho <- seq(0.8, 0.99, by=0.01)

for (i in 1:length(rho)){

modAR<-bam(ppsvc~offset(taxspec)+s(time,k=20, by=seg8pre,bs="cr")+region+seg8pre, method="fREML",data=test, nthreads=2, rho= rho[i], AR.start=arstart)#

aic[i] <-AIC(modAR); reml[i] <- modAR$gcv.ubre

}

rho[reml==min(reml)] # rho=0.98 yields the minimum AIC

# now we use rho=0.98 the GAMM fitted below

# here we don't show model selection descibed in the paper. The model below is the selected model with the lowest AIC

modARp<-bam(ppsvc~s(time,k=20, by=seg8pre,bs="cr")+region+seg8pre, method="fREML",

data=test, nthreads=2, rho= 0.98, AR.start=arstart)

summary(modARp)

## fit the netprice model we used the same structure as above

# model selection also not shown

modARn<-bam(netpricevc~s(time,k=20, by=seg8pre,bs="cr")+region+seg8pre, method="fREML",

data=test, nthreads=2, rho= 0.98, AR.start=arstart)

summary(modARn)

#### trend estimation

#### first get weights based on market share

volvsegmonth <-aggregate(volv ~ seg8pre+monthf, data=test, sum)

volvmonth <-aggregate(volv ~ monthf, data=test, sum)

volvtypemonth <-aggregate(volv ~ typef+monthf, data=test, sum)

dat3 <- merge(test,volvsegmonth,by=c("seg8pre","monthf"))

colnames(dat3)[which(names(dat3) == "volv.y")] <- "volvseg"

colnames(dat3)[which(names(dat3) == "volv.x")] <- "volv"

dat4 <- merge(dat3,volvmonth,by=c("monthf"))

colnames(dat4)[which(names(dat4) == "volv.y")] <- "volvmonth"

colnames(dat4)[which(names(dat4) == "volv.x")] <- "volv"

dat5 <- merge(dat4,volvtypemonth,by=c("typef", "monthf"))

colnames(dat5)[which(names(dat5) == "volv.y")] <- "volvtype"

colnames(dat5)[which(names(dat5) == "volv.x")] <- "volv"

dat5$weight<-dat5$volv/dat5$volvseg # weight: market share of SKU within segment and month

dat5$weight2<-dat5$volv/dat5$volvmonth # weight: market share of SKU within month

dat5$weight3<-dat5$volv/dat5$volvtype # weight: market share of SKU within month and type

test<- dat5

# use price model

gobject<-modARp

# obtain design matrix for estimation which contains all the basis functions and other variables

M<-predict(gobject,newdata=test,type="lpmatrix")

# simulate 1000 samples from posterior distribution of the parameter vector

simcoef <-mvrnorm(n=1000, coef(gobject), gobject$Vp)

simfit<-as.matrix(M)%*% t(simcoef) # this creates 1000 samples from the postererior predictive distribution (1000 sample vectors of price)

# now apply different weights for the different type of trends we want to estimate

simfit.w<- simfit*test$weight # weighted by segment market share

simfit.w.o<- simfit*test$weight2 # weighted by sku market share

simfit.w.t<- simfit*test$weight3 # weighted by type marekt share

# Price per stick (pps) results

# first set up plotting functions

# function to plot overall trend estimated from GAMM

predplot.o <- function(simfit, dset, main="", ylab=NA,wap=FALSE, ylimmi=range(simfit)){

simfit.o <- aggregate(simfit, by=list(test$time),sum) # aggregate - for each of the samples we obtain a weighted overall mean

# note we take the sum here rather than the mean, because the weights are set up so that we obtain the weighted mean

atime.o<-simfit.o[,1]

simfit.o<- simfit.o[,-(1)]### exclude group index

simquant.o<-apply(simfit.o,1,quantile,p=c(0.025,0.5,0.975)) # now take summaries of the distribution for Bayesian confidence interval

par(lwd=2)

plot(atime.o,simquant.o[2,],type="n",xlab="month", ylab=ylab,ylim=ylimmi,xlim=c(1,36),

main=main, axes=FALSE, frame.plot=FALSE)

lines(atime.o,simquant.o[2,],type="l",lty=1)

lines(atime.o,simquant.o[1,],type="l",lty=1)

lines(atime.o,simquant.o[3,],type="l",lty=1)

axis(2); axis(4)

abline(v=1:36,lty=3,col="lightgray")

if ((ylimmi[2]-ylimmi[1]) > 0.3) byl<-0.01 else byl=0.01

print(byl)

abline(h=seq(ylimmi[1],ylimmi[2], by=byl),lty=3,col="lightgray")

axis(1, at=c(1,7,13,19,25,31,36), c("May15", "Nov15" ,"May16" ,"Nov16" ,"May17", "Nov17","Apr18"))

polygon(x=c(13,25,25,13), y=c(ylimmi[1],ylimmi[1],ylimmi[2],ylimmi[2]), col = adjustcolor("orangered", alpha.f = 0.1), border = NA)

abline(v=c(11,22,25,31), col="darkgray") # 16.3.2016 Specific; 8.3.2017 Specific, 20.5.2017 MET, 20.11.2017 MET+specific

resulttab<-cbind(atime.o,t(simquant.o) )

colnames(resulttab) <- c("month", "LL", "Median", "UL")

return(resulttab)

}

# function to plot trends estimated from GAMM by some category (byvar) including gaps between trends of the categories

# this is similar to above predplot() function

predplot <- function(simfit, dset, main="", ylab=NA,wap=FALSE, volmod=FALSE,byvar="seg8pre",xyleg=c(0.2,20),new=TRUE, gap=FALSE, segsub=c("1 FM premium","2 FM midprice","3 FM value","4 FM sub value","5 RYO premium" ,"6 RYO mid price", "7 RYO value","8 no segment"), cigcols= NA){

if (is.na(cigcols)[1]) if (byvar=="seg8pre") cigcols<-c("green", "red", "blue", "purple4","green", "red", "blue", "darkgray") else cigcols<-c("darkblue", "darkred")

if (byvar=="seg8pre"){

simfit<-simfit[dset$seg8pre %in% segsub,]

dset<- dset[dset$seg8pre %in% segsub, ]

subs <-segsub

}

else subs <-levels(dset$typef)

simfit <- aggregate(simfit, by=list(dset$time, dset[,byvar]),sum)

atime<-simfit[,1];

aseg8pre<-simfit[,2]

simfit<-simfit[,-(1:2)] ### exclude group index

# 1. price by segment

simquant<-apply(simfit,1,quantile,p=c(0.025,0.5,0.975))

segc<-c("1FMpremium","2FMmidprice", "3FMvalue", "4FMsubvalue", "5RYOpremium", "6RYOmidprice" ,"7RYOvalue" ,"8nseg")

if (length(subs)<8) ltype=rep(1,length(subs)) else ltype=c(1,1,1,1,2,2,2,2)

par(lwd=2)

ylimmi<- range(simfit); ylimmi[2]<-ylimmi[2]+0.2*(range(simfit)[2]-range(simfit)[1])

if (new) {plot(atime,simquant[2,],type="n",xlab="month", ylab=ylab,ylim=ylimmi,xlim=c(1,36),

main=main, axes=FALSE, frame.plot=FALSE)

axis(2); axis(4)

abline(v=1:36,lty=3,col="lightgray")

abline(h=seq(round(ylimmi[1],2),ylimmi[2], by=0.01),lty=3,col="lightgray")

axis(1, at=c(1,7,13,19,25,31,36), c("May15", "Nov15" ,"May16" ,"Nov16" ,"May17", "Nov17","Apr18"))

polygon(x=c(13,25,25,13), y=c(ylimmi[1],ylimmi[1],ylimmi[2],ylimmi[2]), col =adjustcolor("orangered", alpha.f = 0.1)

, border = NA)

abline(v=c(11,22,25,31), col="darkgray") # 16.3.2016 Specific; 8.3.2017 Specific, 20.5.2017 MET, 20.11.2017 MET+specific

}

j<-0

for (i in subs){

j<-j+1

indi<-aseg8pre==i

lines(atime[indi],simquant[2,indi],type="l",col=cigcols[j],lty=ltype[j])

lines(atime[indi],simquant[1,indi],type="l",col=cigcols[j],lty=ltype[j],lwd=1)

lines(atime[indi],simquant[3,indi],type="l",col=cigcols[j],lty=ltype[j],lwd=1)

}

resulttab<-cbind(atime,t(simquant) )

colnames(resulttab) <- c("month", "LL", "Median", "UL")

rownames(resulttab) <-as.character(aseg8pre)

return(resulttab) # output the actual numbers

}

# overall

tab.overall.pps <- predplot.o(simfit=simfit.w.o, dset=test, main="weighted price per stick", ylab="weighted pps (Â£)", wap=TRUE,ylimmi=c(0.17,0.42))

# by type

tab.fmryo.pps <-predplot(simfit=simfit.w.t, dset=test, main="weighted pps by type", ylab="weighted pps (Â£)", wap=TRUE, byvar="typef", xyleg=c(1, 0.218),new=FALSE, gap=FALSE)

legend(y=0.3,x=1,legend=c("overall", "FM","RYO"), col=c("black","darkblue", "darkred"), lty=1)

# by segments for FM

tab.FM.pps<-predplot(simfit=simfit.w, dset=test, main="weighted price per stick FM", ylab="weighted pps (Â£)", wap=TRUE, byvar="seg8pre", gap=FALSE, segsub=c("1 FM premium","2 FM midprice","3 FM value","4 FM sub value"))

legend(y=0.55,x=1,legend=c("premium","midprice","value","sub value"), col=c("green", "red", "blue", "purple"), lty=1)

# by segments for RYO

tab.RYO.pps<-predplot(simfit=simfit.w, dset=test, main="weighted price per stick RYO", ylab="weighted pps (Â£)", wap=TRUE, byvar="seg8pre", xyleg=c(24, 0.12), gap=FALSE, segsub=c("5 RYO premium" ,"6 RYO mid price", "7 RYO value","8 no segment"),cigcols=c("green", "red", "blue", "darkgray"))

legend(y=0.225,x=1,legend=c("premium" ,"mid price", "value","no segment"), col=c("green", "red", "blue", "darkgray"), lty=1)

# References

1. HMRC. HM Revenue and Customs Value Added Tax (VAT) Bulletin - July 2016 2016 [cited 2016 7th October]. Available from: <https://www.uktradeinfo.com/Statistics/Pages/TaxAndDutybulletins.aspx>.

2. HMRC. HM Revenue and Customs Tobacco Bulletin - July 2016 2016 [cited 2016 7th October]. Available from: <https://www.uktradeinfo.com/Statistics/Pages/TaxAndDutybulletins.aspx>.

3. HMRC. Cigarettes: 'Flash' marks and promotional material: significance for duty calculation. HMRC internal manual tobacco products duty [Internet]. 2016 [cited 2016 6th December]; (TPD7070). Available from: <https://www.gov.uk/hmrc-internal-manuals/tobacco-products-duty/tpd7070>.

4. HMRC. Cigarettes meaning of 'description' or 'brand' for duty purposes. HMRC internal manual tobacco products duty [Internet]. 2016 [cited 2016 6th December]; (TPD7060). Available from: <https://www.gov.uk/hmrc-internal-manuals/tobacco-products-duty/tpd7060>.

5. Lipson F. Budget 2017: Smokers to be hit by new 'floor price' tax on cigarette packs MoneySavingExpert.com2017 [updated 8th March; cited 2018 11th October]. Available from: <https://www.moneysavingexpert.com/news/2017/03/budget-2017-alcohol-and-tobacco-duty-changes/>.

6. Wagner. Segmented regression analysis of interrupted time series studies in medication use research. Journal of Clinical Pharmacy and Therapeutics. 2002.

7. Akaike H. Information theory and an extension of the maximum likelihood principle,[w:] proceedings of the 2nd international symposium on information, bn petrow, f. Czaki, Akademiai Kiado, Budapest. 1973.

8. Wood SN. Package 'mgcv' 2019 [cited 2019 11the August]. Available from: <https://cran.r-project.org/web/packages/mgcv/mgcv.pdf>.

9. Wahba G. Bayesian "Confidence Intervals" for the Cross-Validated Smoothing Spline. Journal of the Royal Statistical Society - Series B (Methodological). 1983;45(1):133–50.

10. Wood SN. Generalized additive models: an introduction 2nd ed: Chapman & Hall/CRC; 2017.

11. Silverman B. Some Aspects of the Spline Smoothing Approach to Non-Parametric Regression Curve Fitting. Journal of the Royal Statistical Society Series B (Methodological) 1985;47(1):1-52.

12. Wood SN, Chapman R, Hall CRC. Generalized additive models: an introduction with R. 2nd ed2017.

13. Augustin N, Mattocks C, Faraway J, Greven S, Ness A. Modelling a response as a function of high-frequency count data: the association between physical activity and fat mass. Statistical methods in medical research. 2017;26(5):2210-26.
